# Alcohol drinking and risks of liver cancer and non-neoplastic chronic liver diseases in China: a 10-year prospective study of 0.5 million adults

**DOI:** 10.1186/s12916-021-02079-1

**Published:** 2021-09-17

**Authors:** Pek Kei Im, Iona Y. Millwood, Christiana Kartsonaki, Yu Guo, Yiping Chen, Iain Turnbull, Canqing Yu, Huaidong Du, Pei Pei, Jun Lv, Robin G. Walters, Liming Li, Ling Yang, Zhengming Chen, on behalf of the China Kadoorie Biobank (CKB) collaborative group

**Affiliations:** 1 Clinical Trial Service Unit and Epidemiological Studies Unit (CTSU), Nuffield Department of Population Health, University of Oxford, Oxford, UK; 2 Medical Research Council Population Health Research Unit (MRC PHRU), Nuffield Department of Population Health, University of Oxford, Old Road Campus, Oxford, OX3 7LF UK; 3 Chinese Academy of Medical Sciences, Beijing, China; 4 Department of Epidemiology and Biostatistics, School of Public Health, Peking University, Beijing, China

**Keywords:** Drinking patterns, Liver cirrhosis, Alcoholic liver disease, Cohort studies

## Abstract

**Background:**

Alcohol consumption is an important risk factor for hepatic neoplastic and non-neoplastic diseases. Questions remain, however, about the relevance to disease risk of drinking patterns and alcohol tolerability, which differ appreciably between Chinese and Western populations.

**Methods:**

The prospective China Kadoorie Biobank included 512,715 adults (41% men) aged 30–79 years recruited from ten areas during 2004–2008, recording alcohol intake, drinking patterns, and other characteristics. After median 10 years’ follow-up, 2531 incident liver cancer, 2040 liver cirrhosis, 260 alcoholic liver disease (ALD), and 1262 non-alcoholic fatty liver disease (NAFLD) cases were recorded among 492,643 participants without prior cancer or chronic liver disease at baseline. Cox regression was used to estimate adjusted hazard ratios (HR) relating alcohol intake and drinking patterns to each disease.

**Results:**

Overall, 33% of men and 2% of women drank alcohol regularly (i.e. at least weekly) at baseline. Among male current regular drinkers, alcohol consumption showed positive dose-response associations with risks of several major chronic liver diseases, with HRs per 280 g/week (i.e. around four drinks/day) higher usual alcohol intake of 1.44 (95% CI 1.23–1.69) for liver cancer (*n* = 547), 1.83 (1.60–2.09) for liver cirrhosis (*n* = 388), 2.01 (1.77–2.28) for ALD (*n* = 200), 1.71 (1.35–2.16) for NAFLD (*n* = 198), and 1.52 (1.40–1.64) for total liver disease (*n* = 1775). The association with ALD appeared stronger among men reporting flushing (i.e., with low alcohol tolerance). After adjustment for the total amount of weekly alcohol consumption, daily drinkers had significantly increased risk of ALD (2.15, 1.40–3.31) compared with non-daily drinkers, and drinking without meals was associated with significantly greater risks of liver cancer (1.32, 1.01–1.72), liver cirrhosis (1.37, 1.02–1.85), and ALD (1.60, 1.09–2.33) compared with drinking with meals. Female current regular drinkers had significantly higher risk of ALD, but not other liver diseases, than female abstainers.

**Conclusions:**

In Chinese men, alcohol intake was associated with significantly increased risks of several major chronic liver diseases, and certain drinking patterns (e.g. drinking daily, drinking without meals) may further exacerbate the disease risks.

**Supplementary Information:**

The online version contains supplementary material available at 10.1186/s12916-021-02079-1.

## Background

Liver disease is a major public health problem worldwide, responsible for > 2 million deaths in 2017, including 1.3 million from liver cirrhosis and > 0.8 million from liver cancer [1, 2]. China is a major contributor to the global burden of liver disease, accounting for over half of worldwide liver cancer cases and deaths, with approximately 300 million people being affected by chronic hepatitis B virus (HBV) infection and other chronic liver diseases [3]. In China, both alcoholic liver disease (ALD) and non-alcoholic fatty liver disease (NAFLD) are emerging as leading causes of chronic liver disease, possibly due to changes in lifestyles along with rapid economic growth [3].

Alcohol consumption is a major risk factor for ALD and liver cirrhosis [4, 5]. Excess risks of ALD and liver cirrhosis have been linked to certain drinking patterns such as drinking without meals and drinking daily in European populations [6, 7]. However, the associations have not yet been extensively investigated in low- and middle-income populations, including China where the main drinking patterns (predominantly drinking spirits, drinking with meals) and genetic tolerability of alcohol (due to a common loss-of-function variant of the *ALDH2* gene, which causes an accumulation of toxic acetaldehyde that leads to the alcohol flushing response after drinking) differ importantly from the West [3, 8, 9]. Previous studies, mainly involving Western populations, suggested that heavy alcohol use may interact with hepatitis C virus (HCV) infection [10, 11] and with certain metabolic risk factors (e.g. obesity, diabetes) [12] to affect the development and progression of chronic liver diseases, but how the associations of alcohol intake with liver diseases might be affected by other factors such as HBV, alcohol tolerability, and adiposity in relatively lean Asian populations remains unclear. Several previous studies in China have reported on the associations of alcohol consumption with risks of liver diseases, but most were conducted decades ago and are further constrained by small numbers of events, limited information on drinking patterns, and single baseline measurement of alcohol consumption [13–16].

To address this evidence gap, we report findings from the nationwide China Kadoorie Biobank (CKB) prospective study of 0.5 million adults. The study aims to (1) assess the associations of alcohol consumption and drinking patterns with incidence of overall and major chronic liver diseases; and (2) examine the associations of alcohol consumption with risks of liver diseases in certain population subgroups (e.g. by HBV infection status, smoking status, body mass index [BMI], prevalent diabetes) and by the alcohol flushing response.

## Methods

### Study design and population

Details of the CKB study design and methods have been previously reported [17]. Briefly, the baseline survey was conducted during 2004–2008 in ten geographically diverse (five urban, five rural) areas across China, selected from China’s nationally representative Disease Surveillance Points (DSP) system to maximise diversity in socioeconomic levels, risk factor exposures and disease patterns, while taking account of population stability and health record system quality. Permanent, non-disabled residents from 100 to 150 rural villages or urban committees in each study area were identified from local residential records and invited to participate. Overall, 512,715 adults aged 30–79 years were enrolled (overall response rate ~ 30%). At local study assessment clinics, trained health workers administered a laptop-based questionnaire recording information on socio-demographic characteristics, alcohol drinking, smoking, diet, physical activity, self-reported health status, and personal and family medical history (e.g. cancer, diabetes); undertook physical measurements (e.g. blood pressure, anthropometry); and collected a blood sample for long-term storage and onsite blood tests including hepatitis B surface antigen (HBsAg) (ACON Biotech) and random plasma glucose level (Johnson & Johnson SureStep Plus Meter). Two resurveys of ~ 5% randomly selected surviving participants were conducted using similar procedures in 2008 and 2013–2014. Ethical approval was obtained from local, national, and international ethical committees. All participants provided written informed consent.

### Assessment of alcohol drinking

Detailed questionnaire assessment of self-reported alcohol consumption patterns has been described previously [9, 18–20]. Based on their past and current drinking history, participants were classified into: (1) abstainers (no alcohol use in the past year and never drank in most weeks in the past); (2) ex-regular drinkers (no or occasional alcohol use in the past year but previously drank in most weeks); (3) occasional drinkers (occasional alcohol use in the past year but never drank in most weeks); and (4) current regular drinkers (some alcohol use in most weeks, i.e. ≥ weekly, in the past year). Current regular drinkers were asked further questions about their drinking patterns including drinking frequency; beverage type and amount of alcohol consumed on a typical drinking day; time of drinking in relation to meals; age started drinking regularly; and experience of the alcohol flushing response (i.e. the experience of hot flushes or dizziness after drinking the first mouthful or a small amount of alcohol). Level of total alcohol intake was calculated as grams (g) of pure alcohol per week, based on the beverage type, amount drunk on a typical day, and frequency. Heavy episodic drinking (HED) was defined as consuming > 60 g of alcohol on a typical drinking occasion for men, and > 40 g per occasion for women [21]. Further details of alcohol assessment are described in Additional file 1: Supplementary Methods and Table S1.

### Follow-up for morbidity and mortality

Cause-specific mortality and morbidity was monitored through local death registries, cancer registries, and the national health insurance claim databases. To minimise loss to follow-up and underreporting of events, annual active follow-up through local residential, health insurance, and administrative records was conducted, supplemented where necessary by contacts with participants and/or their family members. All events were coded using the International Classification of Diseases, 10th Revision (ICD-10). The outcomes studied were incident total liver disease (ICD-10: B18-B19, B94.2, C22, K70-K77, Z22.5) and its main components, including liver cancer (C22), liver cirrhosis (K74), ALD (K70), NAFLD (K76.0), and chronic viral hepatitis (B18-B19, B94.2, Z22.5). By January 1, 2017, 44,037 (8.6%) participants had died and 4781 (0.9%) were lost to follow-up.

### Statistical analysis

Participants with a self-reported history of cancer, liver cirrhosis, or chronic hepatitis at baseline (n=8717), or with unclear or missing HBsAg test results (n=11,355) were excluded, leaving 492,643 participants (201,039 men, 291,604 women) in the main analyses. Given very few women drank alcohol regularly [9, 18], the main analyses were focused on men.

Means and percentages of selected baseline characteristics were calculated across different drinking categories using direct standardisation to the age and study area structure of the study population where appropriate. Cox regression models were used to estimate adjusted hazard ratios (HRs) for incident liver diseases associated with alcohol drinking status (reference category: abstainers) in all participants, and with alcohol consumption level (in categories [reference category: < 140 g/week] and as a continuous variable [per 280 g/week increment, i.e. around four drinks per day]) and with drinking patterns among current regular drinkers. The analyses of the dose-response relationships with amount of alcohol intake and of drinking patterns were focused on current regular drinkers to limit potential biases from reverse causation (e.g. sick-quitter effect where changes in health condition leads to alcohol cessation) and residual confounding (systematic differences in health-related determinants between regular drinkers and non-drinkers as well as occasional drinkers, e.g. long-standing health-related and social factors, some of which are hard to measure) [8, 9, 18, 22, 23]. Cox regression models were stratified by age-at-risk (5-year groups), ten study areas, and HBsAg sero-status (negative vs. positive), and were adjusted for education, household income, smoking, physical activity, and BMI. Analyses of drinking patterns were additionally adjusted for weekly alcohol consumption amount (as a continuous variable), with analyses of drinking duration further adjusted for baseline age.

To account for within-person variation of alcohol intake over the follow-up period [24], repeat alcohol measures for the sample participants who attended both subsequent resurveys were used to estimate usual alcohol intake and to correct for regression dilution bias (see Additional file 1: Supplementary Methods, Table S2). The shapes of associations between amount of alcohol consumption and different liver diseases were assessed by estimating the HRs of predefined baseline consumption categories (< 140, 140–279, 280–419, 420+ g/week in men) and plotting them against the corresponding mean usual alcohol intake. The shapes of associations were also examined using splines. For analyses involving comparisons of just two groups (i.e. an exposure category with the reference group), conventional 95% CIs were reported. For analyses involving more than two exposure categories, floating SEs were used to estimate group-specific 95% CIs of the HRs of all categories including the reference group, enabling comparison between any two categories [25].

To investigate the potential effect modification, HRs per 280 g/week higher usual intake were examined across subgroup populations defined by HBsAg sero-status, smoking status, BMI, flushing response, prevalent diabetes (previously diagnosed or screen-detected at baseline), physical activity, and socio-demographic characteristics (age, study area, education, household income), with heterogeneity in effect sizes assessed by chi-squared tests [26].

Sensitivity analyses included (1) repeated analyses with inclusion of abstainers, occasional drinkers, and ex-regular drinkers; (2) adjustments for further covariates (prevalent diabetes, systolic blood pressure, family history of cancer and diabetes); (3) adjustments for other drinking habits in drinking pattern analyses; and (4) excluding participants with other prior chronic diseases (e.g. cardiovascular disease, diabetes) or poor self-reported health, or the first three years of follow-up.

The fraction of liver diseases attributable to ever-regular drinking (including both ex- and current-regular drinking) in this study population was calculated as P(HR-1)/HR, where P was the proportion of ever-regular drinkers among those who developed the relevant liver disease in CKB [27]. As ex-regular drinkers may have stopped or reduced drinking due to disease onset or changes in health conditions that might have been related to previous heavy drinking [18, 23], we combined ex-regular and current-regular drinkers to estimate the disease burden attributed to ever-regular drinking behaviour throughout lifetime in this study population.

Analysis was done in SAS version 9.4 and R version 3.6.1.

## Results

Among the 492,643 participants, the mean age was 52 (SD 10.7) years, 41% were men, and 56% lived in rural areas, with 3% testing positive for HBsAg at baseline. Among the 201,039 men included in the main analyses, one third (*n* = 66,977) reported drinking alcohol regularly in most weeks (Table 1). In men, abstainers and ex-regular drinkers tended to be older and to report poor health or prior chronic disease than occasional and current regular drinkers. Among male current regular drinkers, compared with moderate drinkers (< 140 g/week), heavier drinkers tended to be rural residents and less educated, and to more often have other unhealthy risk factors such as smoking regularly. Male current regular drinkers consumed on average 284 g of alcohol per week, with 62% drinking daily, 70% drinking spirits, 86% drinking with meals, and 37% engaging in HED in most weeks (Additional file 1: Table S3). Most drinking patterns were positively correlated with total alcohol intake, except drinking with meals which remained consistent across consumption levels. Heavy drinkers were less likely to report flushing after drinking than moderate drinkers (9% vs. 27%, ≥ 420 vs. < 140 g/week). Among the 291,604 women, only 2% (*n* = 5896) were current regular drinkers, and they had much lower consumption (mean 113 g/week) than men (Additional file 1: Tables S4-S5).

**Table 1:** Baseline characteristics of participants by alcohol drinking categories, in men

					Current regular drinkers				
	Overall	Abstainers	Ex-regular drinkers	Occasional drinkers	All current regular	< 140 g/week	140–279 g/week	280–419 g/week	≥ 420 g/week
Number of participants	201,039	40,657	16,990	76,415	66,977	24,171	18,182	12,306	12,318
**Socio-demographic characteristics**									
Mean age, years (SD)	52.9 (10.9)	57.1 (11.1)	56.9 (10.3)	51.0 (10.8)	51.5 (10.2)	51.6 (10.9)	51.7 (10.2)	50.8 (9.6)	50.2 (9.5)
Urban area, %	43.9	31.4	41.7	44.3	50.8	59.3	53.3	48.8	31.7
Educational attainment > 6 years, %	58.2	54.6	57.1	60.9	58.1	61.2	57.5	58.5	54.5
Income > 20000 yuan/year, %	45.7	42.0	45.1	46.7	46.8	47.8	46.3	45.5	46.9
Married, %	92.9	91.3	93.4	93.3	93.3	94.0	93.3	93.3	92.2
**Lifestyle factors**									
Regular smoking, %	61.2	52.6	61.1	57.0	71.8	65.7	73.1	76.5	80.0
Daily fresh fruit consumption, %	22.8	24.7	24.7	25.0	21.1	25.2	20.3	17.7	15.7
Physical activity, mean MET-h/d (SD)	22.0 (15.2)	21.1 (15.0)	20.3 (14.4)	22.5 (15.6)	22.1 (14.8)	21.9 (14.4)	22.2 (14.8)	22.3 (15.3)	21.3 (15.1)
Daily tea drinking, %	40.7	36.0	40.2	37.2	48.4	45.7	47.7	48.7	52.4
**Physical measurements, mean (SD)**									
SBP, mmHg	133.0 (20.1)	132.2 (21.6)	134.3 (21.5)	131.2 (18.8)	134.4 (19.8)	132.2 (18.9)	134.6 (19.8)	135.9 (20.0)	138.0 (20.7)
DBP, mmHg	79.3 (11.4)	78.4 (11.5)	79.9 (11.7)	78.2 (10.9)	80.4 (11.5)	79.2 (11.1)	80.5 (11.5)	81.4 (11.6)	82.4 (11.7)
BMI, kg/m^2^	23.5 (3.3)	23.3 (3.2)	24.0 (3.4)	23.5 (3.2)	23.5 (3.2)	23.4 (3.2)	23.5 (3.2)	23.5 (3.2)	23.6 (3.2)
**Health and medical history, %**^**a**^									
Poor health	8.6	12.1	16.6	7.5	5.7	5.9	5.8	5.4	6.5
Any chronic disease^b^	20.8	24.6	34.6	19.6	16.7	17.9	16.6	16.0	16.5
Coronary heart disease	2.7	3.3	5.4	2.3	2.0	2.2	1.9	1.7	2.2
Stroke or transient ischaemic attack	2.4	3.7	6.2	1.6	1.3	1.4	1.3	1.1	1.3
Prevalent diabetes	5.6	6.7	8.9	5.0	4.7	4.7	4.4	4.9	5.6
Family history of cancer	16.4	14.4	17.9	16.5	17.2	16.9	17.4	17.6	17.7
Family history of diabetes	6.5	6.1	7.8	6.7	6.2	6.5	6.0	5.9	6.3
HBsAg test positive	3.0	3.4	3.6	2.9	3.0	2.8	3.1	3.3	3.1

*SD*, standard deviation; *MET-h/d*, metabolic equivalents of task per hours per day; *SBP*, systolic blood pressure; *DBP*, diastolic blood pressure; *BMI*, body mass index; *HBsAg*, hepatitis B surface antigen

Participants with unclear or missing HBsAg test result, or with self-reported prior cancer, liver cirrhosis, or chronic hepatitis were excluded

Prevalences and means are directly standardised to the age and study area structure of the male study population as appropriate

^a^All self-reported except for prevalent diabetes which included both self-reported and screen-detected diabetes

^b^Chronic diseases included self-reported history of coronary heart disease, stroke, transient ischaemic attack, diabetes, tuberculosis, rheumatoid arthritis, peptic ulcer, emphysema/chronic bronchitis, gallstone/gallbladder disease, and kidney disease

### Alcohol drinking status and risks of liver diseases among all participants

During 5 million person-years of follow-up (median 10.1 years), 9139 individuals (4641 men, 4498 women) developed liver diseases. Among men, the risks for total and most major chronic liver diseases tended to be higher among ex-regular drinkers, lower among occasional drinkers, and broadly similar among current regular drinkers (except for a significantly higher risk of ALD [HR 14.03, 95% CI 7.34–26.83]), when compared with abstainers (Table 2). Among women, no statistically significant associations were observed between alcohol drinking and most liver diseases except a > 6-fold higher risk of ALD in current regular drinkers than abstainers, but the number of cases included was extremely small (Additional file 1: Table S6).

**Table 2:** Adjusted hazard ratios (HRs) for incident liver diseases associated with alcohol drinking status, in men

		Abstainers		Ex-regular drinkers		Occasional drinkers		Current regular drinkers				
	All ***N***	***N***	HR (95% CI)	***N***	HR (95% CI)	***N***	HR (95% CI)	***N***	HR (95% CI)	***P*** value^**a**^	HR (95% CI) per 280 g/week	***P*** trend^**b**^
Liver cancer	1592	365	1.00 (0.90–1.12)	203	1.24 (1.08–1.43)	477	0.86 (0.78–0.94)	547	1.07 (0.98–1.17)	0.364	1.44 (1.23–1.69)	< 0.001
Liver cirrhosis	1098	291	1.00 (0.88–1.14)	112	0.97 (0.81–1.18)	307	0.68 (0.60–0.76)	388	0.96 (0.86–1.07)	0.645	1.83 (1.60–2.09)	< 0.001
Alcoholic liver disease	239	10	1.00 (0.53–1.87)	15	3.72 (2.24–6.20)	14	1.11 (0.65–1.88)	200	14.03 (12.00–16.41)	< 0.001	2.01 (1.77–2.28)	< 0.001
Non-alcoholic fatty liver disease	440	64	1.00 (0.77–1.30)	67	1.73 (1.35–2.22)	111	0.91 (0.75–1.10)	198	1.07 (0.93–1.24)	0.645	1.71 (1.35–2.16)	< 0.001
Chronic viral hepatitis	778	162	1.00 (0.85–1.18)	82	1.30 (1.04–1.62)	308	1.00 (0.89–1.13)	226	0.86 (0.75–0.99)	0.196	1.23 (0.94–1.60)	0.135
Total liver disease	4641	1021	1.00 (0.94–1.07)	513	1.16 (1.06–1.27)	1332	0.83 (0.79–0.88)	1775	1.07 (1.01–1.12)	0.136	1.52 (1.40–1.64)	< 0.001

*HR*, hazard ratio; *CI*, confidence interval; *HBsAg*, hepatitis B surface antigen

Participants with unclear or missing HBsAg test result, or with self-reported prior cancer, liver cirrhosis, or chronic hepatitis were excluded

Cox models are stratified by age-at-risk, study area, and HBsAg, and adjusted for education, household income, smoking, body mass index, and physical activity

^a^*P* for association comparing current regular drinkers vs. abstainers

^b^*P* for alcohol consumption (g/week) modelled as a continuous variable among current regular drinkers

### Amount of alcohol consumption and risks of liver diseases among current regular drinkers

**Fig. 1:**
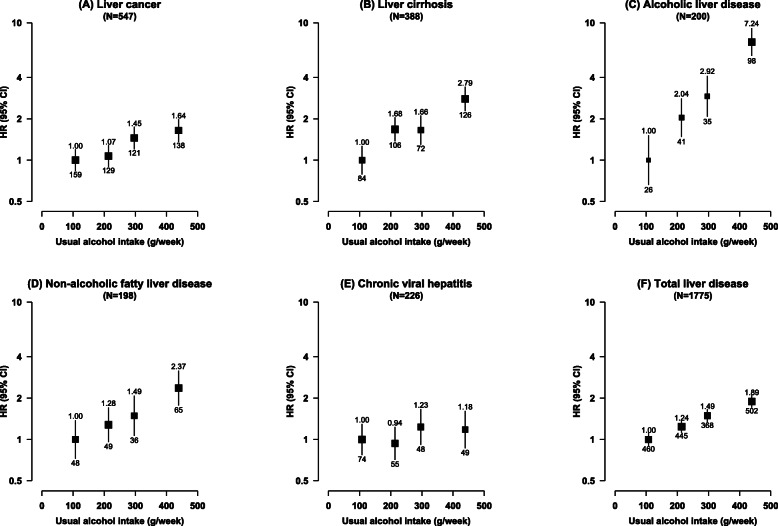
Associations of alcohol consumption with total and major chronic liver diseases, in male current regular drinkers. Cox models are stratified by age-at-risk, study area, and HBsAg, and adjusted for education, household income, smoking, physical activity, and body mass index. Each solid square represents HR with the area inversely proportional to the “floated” variance of the group-specific log hazard. The error bars indicate group-specific 95% CIs. The numbers above the error bars are point estimates for HRs, and the numbers below are number of events. Usual alcohol intake is calculated by the average of the self-reported alcohol intakes of the two resurveys. HBsAg, hepatitis B surface antigen; HR, hazard ratio; CI, confidence interval

Among male current regular drinkers, there were positive dose-response relationships between the usual amount of alcohol intake and risks of total liver disease (HR per 280 g/week 1.52, 95% CI 1.40–1.64), liver cancer (1.44, 1.23–1.69), liver cirrhosis (1.83, 1.60–2.09), ALD (2.01, 1.77–2.28), and NAFLD (1.71, 1.35–2.16), but not chronic viral hepatitis (1.23, 0.94–1.60) (Fig. 1). The shapes of associations were broadly consistent with those observed using spline curves (Additional file 1: Figure S1). Among the few female current regular drinkers, no clear dose-response relationships were observed between the amount of alcohol intake and risks of liver diseases (Additional file 1: Figure S2, Table S7).

**Fig. 2:**
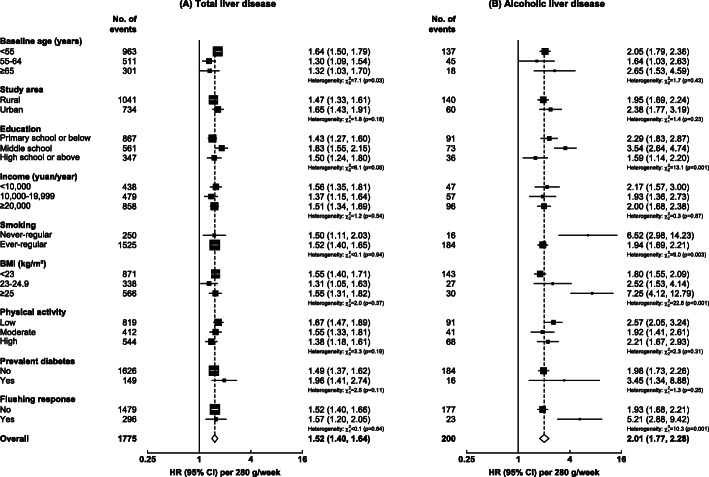
Adjusted HRs per 280 g/week higher usual alcohol intake for total liver disease and alcoholic liver disease, by population subgroups in male current regular drinkers. Cox models are stratified by age-at-risk, study area, and HBsAg, and adjusted for education, household income, smoking, physical activity, and BMI, where appropriate. Each solid square represents HR with the area inversely proportional to the variance of the log HR. The error bars indicate 95% CIs. HBsAg, hepatitis B surface antigen; HR, hazard ratio; CI, confidence interval; BMI, body mass index

Among men, the associations of alcohol intake with total liver disease were broadly consistent across selected population subgroups defined by socio-economic factors, smoking status, BMI, physical activity, diabetes status, and the flushing response (Fig. 2A), which was similar for most major chronic liver diseases (Additional file 1: Figure S3). However, for ALD, the HRs per 280 g/week higher alcohol intake appeared greater among men who never smoked regularly, had higher BMI, or reported flushing after drinking, compared with their counterparts, but these were based on small numbers of events (Fig. 2B). Examination of the joint effects of alcohol and BMI showed that men with higher BMI had consistently higher risks of NAFLD at each alcohol intake level, with > 7-fold higher risk in men who drank ≥ 420 g/week and had a BMI of ≥ 25 kg/m^2^ than those who drank < 140 g/week and had a BMI of < 25 kg/m^2^ (Additional file 1: Figure S4). The risks tended to be lower in men with higher BMI at each alcohol intake level for ALD, albeit with wide CIs, but were otherwise broadly similar across BMI levels for other liver diseases. Participants who tested HBsAg positive had substantially higher risks of liver diseases at all levels of alcohol consumption, although the magnitudes of risk per 280 g/week were similar across HBsAg sero-status (Additional file 1: Figures S5-S6).

### Drinking patterns and risks of liver diseases among current regular drinkers

**Fig. 3:**
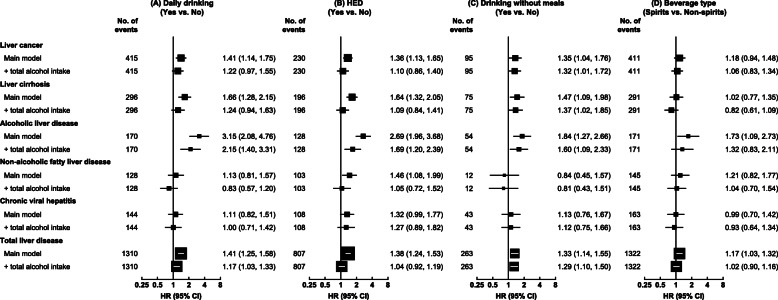
Associations of drinking patterns with total and major chronic liver diseases, in male current regular drinkers. Cox models are stratified by age-at-risk, study area, and HBsAg, and adjusted for education, income, smoking, physical activity, and body mass index, and total alcohol intake where indicated. HED, heavy episodic drinking. Conventions are as in Fig. 2

Among male current regular drinkers, drinking daily and HED were each associated with increased risks of total and major chronic liver diseases, but the associations were greatly attenuated after adjusting for total alcohol consumption (Fig. 3A, B). Given the amount of alcohol consumed, drinking daily was significantly associated with increased risks of ALD (2.15, 1.40–3.31) and total liver disease (1.17, 1.03–1.33), while HED was associated with increased risk of ALD (1.69, 1.20–2.39). When examined across strata of weekly intake level, drinking daily remained associated with higher risks for ALD and total liver disease, although non-significant after further adjustment for total alcohol intake within strata (Additional file 1: Table S8), but there were no clear associations between HED and ALD (Additional file 1: Table S9).

**Fig. 4:**
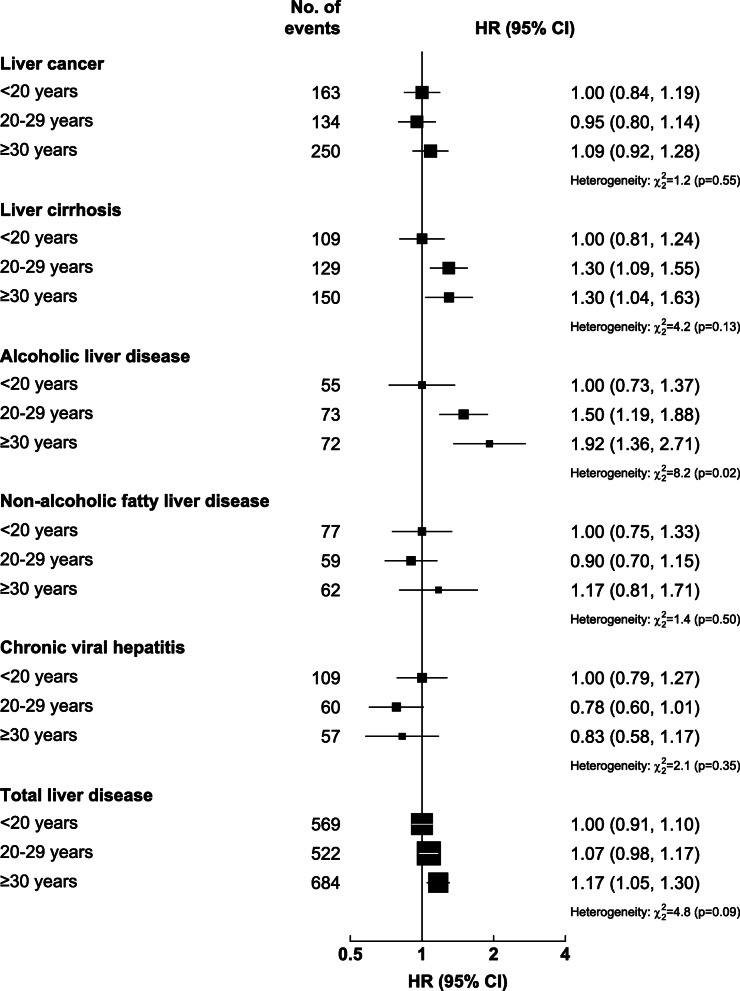
Associations of the duration of regular drinking with total and major chronic liver diseases, in male current regular drinkers. Cox models are stratified by age-at-risk, study area, and HBsAg, and adjusted for education, household income, smoking, physical activity, body mass index, total alcohol intake, and baseline age. Conventions are as in Fig. 1

Similarly among men, drinking without meals was associated with a significantly higher risk of total liver disease than drinking with meals, which persisted after adjustment for total alcohol intake (1.29, 1.10–1.50) (Fig. 3C) and appeared more evident at higher intake levels (Additional file 1: Figure S7), and with 32–60% higher risks of liver cancer, liver cirrhosis, and ALD. The risks of liver diseases did not differ between spirits and other beverage drinkers (i.e. beer, rice wine and grape wine combined) given the amount consumed (Fig. 3D, Additional file 1: Figure S8). Adjusting for total alcohol intake and baseline age, a longer duration of regular drinking was associated with progressively higher risks of total liver disease and ALD, but not other liver diseases (Fig. 4). Having the alcohol flushing response per se was associated with lower ALD risk than not flushing, but the association became non-significant after adjustment for total alcohol intake (Additional file 1: Table S10).

### Sensitivity analyses and attributable risk

The dose-response associations among male current regular drinkers were similar with further adjustments (prevalent diabetes, blood pressure, family history of cancer and diabetes) (Additional file 1: Table S11), and the associations for drinking patterns were generally unaffected by further adjustments for other drinking habits (Additional file 1: Tables S12-S15). Inclusion of abstainers, occasional drinkers and ex-regular drinkers in the analyses showed an overall J-shaped association of alcohol drinking with most liver diseases (Additional file 1: Figure S9). Excluding the first three years of follow-up and participants with prior chronic disease or poor self-reported health tended to increase the HRs in regular drinkers for most liver diseases and in occasional drinkers for ALD and chronic viral hepatitis while attenuating the lower HRs in occasional drinkers for other liver diseases, compared with abstainers (Additional file 1: Table S16), but did not alter the dose-response relationships among current regular drinkers (Additional file 1: Table S17).

If the observed associations between alcohol consumption and liver diseases are largely causal, approximately 9% of all incident liver cancer (8.5%, 95% CI 4.3–12.7%) and non-neoplastic liver diseases (8.7%, 5.7–11.7%), respectively, in the male study population could be attributed to ever-regular alcohol drinking (Additional file 1: Table S18).

## Discussion

In this large prospective study, current regular alcohol drinking was associated with significantly increased risks of liver cancer and several non-neoplastic chronic liver diseases in Chinese men, with significant dose-response relationships between alcohol intake and risks of liver cancer, liver cirrhosis, ALD, and NAFLD. The associations were consistent across subgroups defined by other risk factors (e.g. smoking, BMI, HBV infection, diabetes), although there was a suggestion of stronger associations of alcohol intake with ALD among those who had higher BMI, or who had the alcohol flushing response. For a given level of weekly intake, drinking daily and having a longer duration of regular drinking were associated with excess risks of ALD, while drinking without meals was associated with excess risks of liver cancer, liver cirrhosis, and ALD. Among the few female current regular drinkers, the risk of ALD was significantly elevated compared with abstainers, but the associations with other disease types were less clear.

A J-shaped association of alcohol consumption with chronic liver disease has been observed in previous prospective studies of Western populations [28, 29], with similar findings also shown in the present study of Chinese adults when the analyses included all alcohol drinking categories. These associations are likely to be affected by reverse causality and confounding, as pre-existing poor health or social disadvantages may lead to alcohol cessation or abstinence and, indeed, in our study abstainers and ex-regular drinkers tended to be older and had poorer baseline health. Furthermore, removing early follow-up years and participants with poor baseline health attenuated the apparently “protective” effects of occasional drinking compared with not drinking but increased the risks associated with regular drinking. To help reliably assess the dose-response relationships between the amount of alcohol intake and liver diseases, we focused the main analyses among male current regular drinkers and showed significant dose-response associations between alcohol consumption and total liver disease incidence as well as mortality in Chinese men (Additional file 1: Tables S19-S20), consistent with previous studies in high-income populations [28, 30].

Although alcohol consumption has been concluded to be causally related to liver cancer by WHO-IARC [31], evidence from China, where liver cancer has been historically primarily related to chronic HBV infection [3], is limited. A meta-analysis of 18 case-control studies from China reported a significant odds ratio of 1.56 for hepatocellular carcinoma in drinkers versus non-drinkers (3812 cases) [32], but no clear dose-response relationships were reported in prospective studies, which were conducted almost three decades ago when alcohol consumption was lower in China [13, 14, 16]. In our study, which was conducted in more recent decades, there were clear dose-response relationships between alcohol intake and liver cancer risk in Chinese men, largely independent of HBV infection status.

Alcohol intake has been consistently shown to be associated with increased risks of liver cirrhosis and ALD in Western and high-income Asian populations [4–7, 30]. In China, previous prospective studies have also reported excess liver cirrhosis mortality in male heavy drinkers [13, 15]. With linkage to the national health insurance system which covered almost all hospitalised events, our study now provides reliable prospective evidence of significant dose-response relationships between alcohol intake and incident liver cirrhosis and ALD in Chinese men.

In populations with high prevalence of obesity, fatty liver disease particularly NAFLD has emerged as a major chronic liver disease. Two meta-analyses of 18 and 16 individual studies, respectively, suggested that moderate drinking (i.e. < 20 g/day) was associated with lower risk of fatty liver disease (diagnosed and defined in various ways across different studies) compared with non-drinking [33, 34]. However, these findings were based mostly on cross-sectional studies and were inconsistent across different populations. Conversely, the longitudinal Whitehall II Study (*n* = 5407, 30-year follow-up) reported a twofold higher risk of fatty liver disease (defined as having a fatty liver index ≥ 60) associated with sustained heavy drinking versus stable low-risk drinking (> 168 vs. 8–168 g/week for men, > 112 vs. 8–112 g/week for women), with no reduced risks for stable low-risk drinking compared with stable non-drinking [35]. With a much larger size of study population than previous studies, we provide new prospective evidence for a positive dose-response relationship between alcohol intake and NAFLD in Chinese male drinkers. Unlike some of the previous studies, the diagnosis of NAFLD in the present study was based on hospital admission rather than ultrasound test and there were strong positive associations between NAFLD and adiposity. Moreover, the diagnosis of NAFLD did not overlap with ALD (with less than ten having a diagnosis of both diseases during follow-up), suggesting the finding was unlikely to be due to misclassification of ALD.

Several possible mechanisms linking alcohol consumption with different types of liver diseases have been proposed [36]. Alcohol use, particularly heavy consumption, can stimulate changes in lipid metabolism resulting in fatty liver. Moreover, heavy alcohol consumption may induce an inflammatory response resulting in alcoholic hepatitis (or steatohepatitis if accompanied by hepatic lipid deposition) which, if persistent and severe, can eventually lead to fibrosis and sclerotic changes in the liver resulting in liver cirrhosis, and ultimately liver failure or liver cancer. Previous studies showed that heavy alcohol consumption may interact with chronic HCV infection, possibly via increased viral replication and altered immune response, to aggravate liver disease progression and increase mortality risk [10, 11, 37]. Similar pathways have been assumed for HBV infection [37], but there was no clear evidence of interaction between alcohol consumption and HBV infection on risks of liver diseases in our study.

It has been suggested that smoking [29], obesity [38, 39], and diabetes [40] may synergistically interact with alcohol consumption to increase the risks of advanced liver diseases, but the existing epidemiological evidence is inconsistent [28–30, 38–40]. In our study, although the associations of alcohol consumption with most major chronic liver diseases appeared consistent between subgroups defined by these risk factors, stronger associations of alcohol intake with ALD were observed in never smokers, men with higher BMI, or men reporting flushing, compared with their counterparts. However, due to the limited number of ALD events, these findings should be interpreted with caution. It is possible that obesity and alcohol consumption may synergistically worsen hepatic insulin resistance and cause necro-inflammation, leading to progressive liver injury [41]. In animal studies, aldehyde dehydrogenase 2 (ALDH2) deficiency has been shown to aggravate liver inflammation and fibrosis through increased exposure to acetaldehyde and its associated adducts after alcohol consumption [42]; although epidemiological studies reported a reduced alcoholic cirrhosis risk in individuals with ALDH2 deficiency [43], this is likely due to the substantially lower alcohol consumption in individuals with ALDH2 deficiency which inhibits heavy drinking.

This is the first prospective study to report on the associations between drinking patterns and risks of many different types of chronic liver diseases in a Chinese population. The UK Million Women Study (MWS) and the Danish Diet, Cancer and Health Study have shown that daily drinking was associated with increased risks of liver cirrhosis and ALD compared with non-daily drinking (HR range 1.61–3.65), given the amount consumed [6, 7]. Two small studies in Finland and the US (< 130 cases each) have also reported positive associations between HED frequency and risks of liver diseases (HR range 2.78–4.76 for ≥ weekly vs. never/rare engagement in HED), but the findings were prone to residual confounding by the total volume and frequency of alcohol consumption [44, 45]. Our findings seem to support the adverse effects of daily drinking on ALD and total liver disease over and above total alcohol intake. There may be increased hepatic damage with consistent exposure to alcohol, compared with taking breaks from drinking (i.e. “liver holidays”) which allows recovery of the normal metabolic function of the liver [46]. Nevertheless, the findings should not be taken to dispute the potential harmful effects of HED, as HED results in a high amount consumed and has both acute (e.g. injuries, acute alcohol poisoning) and long-term consequences (e.g. hypertension, alcohol dependence), and chronic persistent HED can exacerbate liver injury [47]. Our findings in men were generally consistent with those reported in the MWS, which showed a 31% lower risk of liver cirrhosis and ALD (1560 cases) among women who usually drank alcohol with meals than those who did not [6]. In the absence of food, there may be faster absorption of alcohol from the intestine, leading to higher blood alcohol concentrations and greater detrimental effects of drinking [48]. Our observed associations between duration of regular drinking and risks of ALD and total liver disease also concur with previous studies [49, 50].

The chief strengths of this study include the prospective study design with a large sample size, comprehensive adjustments for potential confounders, and large numbers of well-characterised liver disease events traced via comprehensive follow-up systems. The exclusions of individuals with prior diseases and early follow-up reduced reverse causality. Also, the repeat alcohol measures allowed adjustment for regression dilution bias [24]. Our study also has several limitations. First, alcohol exposure data was self-reported, but they were positively correlated with blood pressure and gamma-glutamyl transferase as expected and consistent with the causal associations seen with genotype-predicted alcohol intake [8], suggesting good data quality. It is still possible, however, that heavy drinking may be under-reported, which could potentially lead to underestimation of the HED-associated disease risk. Second, it is possible that the observed association with NAFLD and other liver diseases may partly be due to some ALD cases being misclassified as NAFLD (e.g. the patient’s alcohol intake was under-reported or not stated in clinical notes); however, the strong positive associations between NAFLD and adiposity and lack of overlap in different disease diagnoses during follow-up suggested that our findings were unlikely to be due to misclassification of ALD. Third, although careful adjustments were made, residual confounding by measured or unmeasured factors might remain. For example, measurement error in baseline alcohol intake may lead to inadequate adjustment for total alcohol intake in the associations of drinking patterns with disease risks. Where possible, subgroup analyses were performed to further minimise residual confounding. Fourth, although the associations between alcohol consumption and liver diseases differed little by HBsAg sero-status, the on-site HBsAg rapid test used in CKB has relatively low sensitivity for lower serum HBsAg levels [51, 52]. Fifth, the lack of information on HCV infection precluded adjustment for HCV infection in our analyses and investigation of potential interactions between alcohol consumption and HCV infection on disease risks; however, given the low prevalence of HCV infection in China (< 1%) [53] it should not have materially affected our main findings. Sixth, it is possible that flushing response may diminish in intensity after a long or heavy drinking history, potentially leading to some misclassifications which might underestimate any heterogeneity in alcohol-liver disease risk relations by ALDH2 deficiency. Nevertheless, overall the flushing response was strongly associated with *ALDH2*-rs671 genotype among current regular drinkers in CKB [8]. Finally, given the low prevalence of grape wine drinkers in China [9, 18], we were unable to investigate the separate associations for grape wine, which has been reported to have less detrimental effects on alcoholic liver cirrhosis than other beverage types [7]. Likewise, we lacked sufficient statistical power for analyses in women and on ALD across subgroups.

## Conclusions

In summary, among Chinese men, alcohol consumption was positively associated with risks of major chronic liver diseases, including liver cancer, ALD, liver cirrhosis, and NAFLD. As well as total amount consumed, certain drinking patterns especially drinking without meals, drinking daily, and prolonged duration of regular drinking might be particularly harmful. While the mandatory HCV screening for blood transfusion since the early 1990s and the universal HBV immunisation programme among new-born babies since the early 2000s, respectively, are expected to have huge impacts on current and future rates of infection-related liver diseases in China [3], tackling the co-emerging burden of alcohol consumption and obesity becomes a priority in liver disease prevention. Our study provides important new evidence for China and elsewhere that reducing population-levels of alcohol consumption is an important preventive strategy for liver diseases, and certain drinking habits such as drinking daily and drinking without meals should also be discouraged.

## Supplementary Information

**Additional file 1.**

## Data Availability

The China Kadoorie Biobank (CKB) is a global resource for the investigation of lifestyle, environmental, blood biochemical and genetic factors as determinants of common diseases. The CKB study group is committed to making the cohort data available to the scientific community in China, the UK and worldwide to advance knowledge about the causes, prevention and treatment of disease. For detailed information on what data is currently available to open access users and how to apply for it, visit: http://www.ckbiobank.org/site/Data+Access. Researchers who are interested in obtaining the raw data from the China Kadoorie Biobank study that underlines this paper should contact ckbaccess@ndph.ox.ac.uk. A research proposal will be requested to ensure that any analysis is performed by bona fide researchers and—where data is not currently available to open access researchers—is restricted to the topic covered in this paper.

## Abbreviations

HBV: Hepatitis B virus
ALD: Alcoholic liver disease
NAFLD: Non-alcoholic fatty liver disease
CKB: China Kadoorie Biobank
BMI: Body mass index
DSP: Disease Surveillance Points
HBsAg: Hepatitis B surface antigen
HCV: Hepatitis C virus
HED: Heavy episodic drinking
ICD-10: International Classification of Diseases, 10^th^ Revision
HR: Hazard ratio
WHO: World Health Organization
IARC: International Agency for Research on Cancer
ALDH2: Aldehyde dehydrogenase 2
MWS: Million Women Study
