# Supplementary material for: Alcohol drinking and risks of liver cancer and non-neoplastic chronic liver diseases in China: a 10-year prospective study of 0.5 million adults
Source: BMC Med. 2021 Sep 17;19:216. doi: 10.1186/s12916-021-02079-1 (PMC8447782; doi:10.1186/s12916-021-02079-1)
Supplement: Supplementary file 1 — Additional file 1. [file 12916_2021_2079_MOESM1_ESM.pdf]

Additional File 1

Alcohol drinking and risks of liver cancer and non-neoplastic chronic liver diseases in China: a 10-year prospective study of 0.5 million adults

Pek Kei Im, Iona Y Millwood, Christiana Kartsonaki, Yu Guo, Yiping Chen, Iain Turnbull, Canqing Yu, Huaidong Du, Pei Pei, Jun Lv, Robin G Walters, Liming Li, Ling Yang, Zhengming Chen, on behalf of the China Kadoorie Biobank (CKB) collaborative group

Content List of Supplementary Materials

Members of the China Kadoorie Biobank collaborative group 3

Supplementary Methods 4

Table S1. Definitions of main alcohol drinking patterns 8

Table S2. Estimated regression dilution ratio of alcohol consumption in men and women 9

Table S3. Baseline drinking characteristics of participants by level of alcohol consumption, in male current regular drinkers 10

Table S4. Baseline characteristics of participants by alcohol drinking categories, in women 11

Table S5. Baseline drinking characteristics of participants by level of alcohol consumption, in female current regular drinkers 12

Table S6. Adjusted HRs for total and major chronic liver diseases associated with drinking status, in women 13

Figure S1. Spline curves of adjusted HRs for total and major chronic liver diseases associated with alcohol intake over the range of 0-1500 g/week, in male current regular drinkers 14

Figure S2. Association of alcohol consumption with total liver disease, in female current regular drinkers 15

Table S7. Adjusted HRs for total and major chronic liver diseases associated with amount of alcohol consumption, in female current regular drinkers 16

Figure S3. Adjusted HRs per 280g/week higher usual alcohol intake for liver cancer, liver cirrhosis and non-alcoholic fatty liver disease, by population subgroups in male current regular drinkers 17

Figure S4. Associations of the joint effects of alcohol consumption and body mass index with total and major chronic liver diseases, in male current regular drinkers 18

Figure S5. Associations of the joint effects of alcohol consumption and HBsAg seropositivity with total and major chronic liver diseases, in male current regular drinkers 19

Figure S6. Adjusted HRs per 280 g/week higher usual alcohol intake for total and major chronic liver diseases, by HBsAg seroprevalence in male current regular drinkers 20

Table S8. Adjusted HRs for total and major chronic liver diseases associated with daily drinking, by weekly alcohol intake level in male current regular drinkers 21

Table S9. Adjusted HRs for total and major chronic liver diseases associated with heavy episodic drinking, by weekly alcohol intake level in male current regular drinkers 22

Figure S7. Associations of the joint effects of alcohol consumption and mealtime drinking habits with total and major chronic liver diseases, in male current regular drinkers 23
Figure S8. Adjusted HRs per 280 g/week higher usual alcohol intake for total and major chronic liver diseases, by beverage type in male current regular drinkers 24
Table S10. Adjusted HRs for total and major chronic liver diseases associated with the flushing response, in male current regular drinkers 25
Table S11. Adjusted HRs per 280 g/week higher usual alcohol intake for total and major chronic liver diseases, in male current regular drinkers – in sequentially adjusted models 26
Table S12. Adjusted HRs for total and major chronic liver diseases associated with daily drinking, in male current regular drinkers - in sequentially adjusted models 27
Table S13. Adjusted HRs for total and major chronic liver diseases associated with heavy episodic drinking, in male current regular drinkers - in sequentially adjusted models.....................................28
Table S14. Adjusted HRs for total and major chronic liver diseases associated with mealtime drinking habits, in male current regular drinkers - in sequentially adjusted models 29
Table S15. Adjusted HRs for total and major chronic liver diseases associated with beverage type, in male current regular drinkers - in sequentially adjusted models 30
Figure S9. Associations of alcohol consumption with total and major chronic liver diseases, in all men...31
Table S16. Adjusted HRs for total and major chronic liver diseases associated with alcohol drinking status, in men after various exclusions of participants 32
Table S17. Adjusted HRs for total and major chronic liver diseases associated with amount of alcohol consumption, in male current regular drinkers after various exclusions of participants 33
Table S18. Attributable fraction of liver diseases from ever-regular drinking in men in the CKB.....34
Table S19. Adjusted HRs for liver disease mortality associated with drinking status, in men 35
Table S20. Adjusted HRs for liver disease mortality associated with level of alcohol consumption, in male current regular drinkers...36
References...37

Members of the China Kadoorie Biobank collaborative group

International Steering Committee: Junshi Chen, Zhengming Chen (PI), Robert Clarke, Rory Collins, Yu Guo, Liming Li (PI), Chen Wang, Jun Lv, Richard Peto, Robin Walters.

International Co-ordinating Centre, Oxford: Daniel Avery, Fiona Bragg, Derrick Bennett, Ruth Boxall, Ka Hung Chan, Yumei Chang, Yiping Chen, Zhengming Chen, Johnathan Clarke; Robert Clarke, Huaidong Du, Zammy Fairhurst-Hunter, Hannah Fry, Wei Gan, Simon Gilbert, Alex Hacker, Parisa Hariri, Mike Hill, Michael Holmes, Pek Kei Im, Andri Iona, Maria Kakkoura, Christiana Kartsonaki, Rene Kerosi, Kuang Lin, Mohsen Mazidi, Iona Millwood, Qunhua Nie, Alfred Pozarickij, Paul Ryder, Sam Sansome, Dan Schmidt, Paul Sherliker, Rajani Sohoni, Becky Stevens, Iain Turnbull, Robin Walters, Lin Wang, Neil Wright, Ling Yang, Xiaoming Yang, Pang Yao.

National Co-ordinating Centre, Beijing: Yu Guo, Xiao Han, Can Hou, Chun Li, Chao Liu, Jun Lv, Pei Pei, Canqing Yu.

10 Regional Co-ordinating Centres:

Guangxi Provincial CDC: Naying Chen, Duo Liu, Zhenzhu Tang. Liuzhou CDC: Ningyu Chen, Qilian Jiang, Jian Lan, Mingqiang Li, Yun Liu, Fanwen Meng, Jinhuai Meng, Rong Pan, Yulu Qin, Ping Wang, Sisi Wang, Liuping Wei, Liyuan Zhou. **Gansu** Provincial CDC: Caixia Dong, Pengfei Ge, Xiaolan Ren. Maiji CDC: Zhongxiao Li, Enke Mao, Tao Wang, Hui Zhang, Xi Zhang. **Hainan** Provincial CDC: Jinyan Chen, Ximin Hu, Xiaohuan Wang. Meilan CDC: Zhendong Guo, Huimei Li, Yilei Li, Min Weng, Shukuan Wu. **Heilongjiang** Provincial CDC: Shichun Yan, Mingyuan Zou, Xue Zhou. Nangang CDC: Ziyan Guo, Quan Kang, Yanjie Li, Bo Yu, Qinai Xu. **Henan** Provincial CDC: Liang Chang, Lei Fan, Shixian Feng, Ding Zhang, Gang Zhou. Huixian CDC: Yulian Gao, Tianyou He, Pan He, Chen Hu, Huarong Sun, Xukui Zhang. **Hunan** Provincial CDC: Biyun Chen, Zhongxi Fu, Yuelong Huang, Huilin Liu, Qiaohua Xu, Li Yin. Liuyang CDC: Huajun Long, Xin Xu, Hao Zhang, Libo Zhang. **Jiangsu** Provincial CDC: Jian Su, Ran Tao, Ming Wu, Jie Yang, Jinyi Zhou, Yonglin Zhou. Suzhou CDC: Yihe Hu, Yujie Hua, Jianrong Jin Fang Liu, Jingchao Liu, Yan Lu, Liangcai Ma, Aiyu Tang, Jun Zhang. **Qingdao** Qingdao CDC: Liang Cheng, Ranran Du, Ruqin Gao, Feifei Li, Shanpeng Li, Yongmei Liu, Feng Ning, Zengchang Pang, Xiaohui Sun, Xiaocao Tian, Shaojie Wang, Yaoming Zhai, Hua Zhang, Licang CDC: Wei Hou, Silu Lv, Junzheng Wang. **Sichuan** Provincial CDC: Xiaofang Chen, Xianping Wu, Ningmei Zhang, Weiwei Zhou. Pengzhou CDC: Xiaofang Chen, Jianguo Li, Jiaqiu Liu, Guojin Luo, Qiang Sun, Xunfu Zhong. **Zhejiang** Provincial CDC: Weiwei Gong, Ruying Hu, Hao Wang,Meng Wan, Min Yu. Tongxiang CDC: Lingli Chen, Qijun Gu, Dongxia Pan， Chunmei Wang, Kaixu Xie, Xiaoyi Zhang.

Supplementary Methods

Assessment of alcohol consumption

Detailed questionnaire assessment of alcohol consumption has been described previously.[1-4] At the baseline questionnaire, participants were asked how often they had drunk alcohol during the past 12 months (never or almost never, occasionally, only at certain seasons, every month but less than weekly, usually at least once a week). Those who had not drunk alcohol at least weekly in the past 12 months were asked if there was a period of at least a year prior to that when they had drunk some alcohol at least once a week. For this report, participants were classified into four main drinking categories based on the frequency of alcohol drinking during the past 12 months and prior to the past year: (1) abstainers – who had no alcohol use in the past 12 months and had never drunk in most weeks in the past; (2) ex-regular drinkers – who had none or occasional (i.e., occasionally, only at certain seasons, every month but less than weekly) alcohol use in the past 12 months but had previously drunk in most weeks; (3) occasional drinkers – who had occasional alcohol use in the past 12 months but had never drunk in most weeks; and (4) current regular drinkers – who had some alcohol use in most weeks (i.e., ≥weekly) in the past 12 months.

Those who had drunk alcohol at least once a week in the past 12 months (i.e., current regular drinkers) were asked further questions about their drinking patterns including: frequency of drinking in the past year (1-2, 3-5, or 6-7 days per week); type of beverage (beer, grape wine, rice wine, weak spirits with <40% alcohol content, strong spirits with ≥40% alcohol content) and amount consumed for the reported beverage type (reported by number of small [250 ml] or large [640 ml] bottles of beer, and number of liang [50g] for wines and spirits) on a typical drinking day; time of drinking in relation to meals on a typical drinking day (usually drinking with meal, usually drinking between or after the meals, no regular patterns); age started drinking in most weeks; and whether the participant

usually experienced hot flushes or dizziness after drinking alcohol (soon after the first mouthful, after drinking a small amount of alcohol, after drinking a large amount of alcohol, no flushing).

Level of alcohol consumption on a typical drinking day was calculated as grams of pure alcohol, based on the beverage type and amount drunk on a typical drinking day, assuming the following alcohol content by volume (v/v) typically seen in China:[5] beer 4%, grape wine 12%, rice wine 15%, weak spirits 38%, and strong spirits 53%. Total level of weekly alcohol consumption was calculated as grams per week by multiplying the consumption on a typical drinking day by the frequency of drinking (taken as the median of the reported frequency intervals, i.e., 1.5 for 1-2 days/week, 4 for 3-5 days/week, 6.5 for 6-7 days/week). Detailed definitions of the various parameters of alcohol consumption are shown in **Table S1**.

Main analytic models

Cox regression models were used to estimate the associations of alcohol drinking status (reference group: abstainers) with incident liver diseases in all men and women separately, and of alcohol consumption level (in categories [reference group: <140 g/week for men; <70 g/week for women] and as a continuous variable [per 280 g/week higher alcohol intake, i.e., around four drinks per day]) with incident liver diseases in male current regular drinkers and female current regular drinkers separately. Cox regression models were stratified by age-at-risk (five-year groups), ten study areas, and HBsAg sero-status (negative vs. positive), and were adjusted for education (no formal school, primary school, middle or high school, technical school/college or above), household income (<10,000, 10,000-19,999, 20,000-34,999, ≥35,000 yuan/year), smoking (five groups in men: never, occasional, ever regular <15, ever regular 15-24, ever regular ≥25 cigarettes equivalent/day; four groups in women: never, occasional, ex-regular, current), physical activity (<17.8, 17.8-28.7, ≥28.8 metabolic equivalent of task hours [MET-h] per day), and BMI (<22, 22-24.9, 25-26.9, ≥27 kg/m^2). The associations of drinking patterns (daily drinking, HED, mealtime habit, spirit drinking, duration

of regular drinking) with liver diseases were assessed among male current regular drinkers, adjusting for the same covariates plus total weekly consumption (as a continuous variable), and additionally for baseline age (as a continuous variable) in the analyses of duration of regular drinking. For daily drinking and HED, the risk estimates were further examined across subgroups defined by total weekly intake (<280 vs. ≥280 g/week). The proportional hazards assumption for the Cox model was checked using scaled Schoenfeld residuals and by examining the HRs for the first 5 years and for subsequent years of follow-up (no strong evidence of departure from the proportional hazard assumption). For two-way comparisons of exposure categories, conventional SEs and CIs were used. For analyses involving more than two exposure categories, floating SEs were used for the log HRs of all categories including the reference group, enabling comparison between any two categories.[6]

Adjustment for regression dilution bias

A gradual regression to the mean over time was observed for alcohol consumption in the subset of participants involved in all three CKB surveys (**Table S2**). Within-person variation of self-reported alcohol intake was addressed using the regression dilution approach,[7] whereby the usual alcohol intake was taken to be the average intake of the two resurveys in 2008 and 2013-2014, assuming that occasional drinkers consumed 5 g/week. To assess the shape of associations between amount of alcohol intake and liver diseases, the HRs of baseline consumption categories were plotted against their corresponding mean usual alcohol intake. The regression dilution ratio (RDR) was calculated using the assumption-free, non-parametric McMahon-Peto method,[8] taken as the ratio of the range (i.e., difference between the mean alcohol intake of the top [i.e., ≥420 g/week for men; ≥140 g/week for women] vs. bottom [i.e., <140 g/week for men; <70 g/week for women] baseline-defined groups) of the usual alcohol intake levels to the range of the baseline alcohol intake levels. The RDRs calculated using the McMahon-Peto method were 0.54 for men and 0.60 for women, broadly similar to the estimates obtained from other methods, e.g., self-correlation[7] and the Rosner's regression method.[9] Log HR estimates and the corresponding SEs for baseline alcohol intake, modelled as a

continuous variable, were then divided by the RDR calculated from the McMahon-Peto method to obtain estimated HRs per 280 g/week higher usual alcohol intake among current regular drinkers. The HR per 100 g/week is approximately the cube root of the HR per 280 g/week (as log HR per 100 g/week is [100/280] times log HR per 280 g/week).

Subgroup and sensitivity analyses

The HRs per 280 g/week were examined across subgroups defined by HBsAg sero-status (negative vs. positive), smoking status (ever-regular [i.e., ex-regular or current regular smokers] vs. never-regular [i.e., never or occasional smokers]), BMI (<23, 23-24.9, ≥25 kg/m^2), flushing response (yes vs. no), prevalent diabetes (yes vs. no), physical activity (<17.8, 17.8-28.7, ≥28.8 MET-h/day), and socio-demographic characteristics including baseline age (<55, 55-64, ≥65 years), study area (rural vs. urban), education level (primary school or below, middle school, high school or above), and household income (<10,000, 10,000-19,999, ≥20,000 yuan/year). Chi-squared tests were used to assess heterogeneity across strata. Prevalent diabetes included both self-reported, previously diagnosed diabetes and screen-detected diabetes (defined as having a measured random plasma glucose level ≥7.0mmol/L with time since last food intake ≥8 hours, or ≥11.1mmol/L with time since last food intake <8 hours, or a fasting plasma glucose level ≥7.0mmol/L on subsequent testing).[10] Analyses on drinking status and usual amount of alcohol intake were repeated with liver disease mortality for comparison with the main analyses with incident liver diseases. Sensitivity analyses included: 1) further covariate adjustments (for family history of cancer, prevalent diabetes, systolic blood pressure, family history of diabetes) in the analyses of usual alcohol intake; 2) simultaneous adjustments for other drinking habits in drinking pattern analyses; and 3) exclusions of participants with other prior major chronic disease (including self-reported coronary heart diseases, stroke, transient ischaemic attack, tuberculosis, rheumatoid arthritis, peptic ulcer, emphysema/bronchitis, gallstone/gallbladder disease, kidney disease, and prevalent diabetes) or poor self-reported health at baseline, or the first three years of follow-up.

Table S1. Definitions of main alcohol drinking patterns

Parameters	Description
**Drinking status**	
Abstainers	Past 12 months: Never drank alcohol.<br>In the past: Had not drunk weekly or more frequently.
Ex-regular drinkers	Past 12 months: Never drank alcohol; or had drunk alcohol occasionally, at certain seasons, or monthly but less than weekly.<br>In the past: Had drunk at least weekly.
Occasional drinkers	Past 12 months: Had drunk alcohol occasionally, at certain seasons, or monthly but less than weekly.<br>In the past: Had not drunk at least weekly.
Current regular drinkers	Past 12 months: At least weekly (i.e., drank alcohol in most weeks).<br>In the past: N/A.
**Amount of alcohol consumed and drinking patterns among current regular drinkers (i.e., drank ≥weekly in the past 12 months)**	
Weekly intake (g per typical drinking week)	Calculated based on the beverage type[a] and amount drunk[a] on a typical drinking day, and drinking frequency in the past year.<br>**In categories:**<br>Men: <140; 140-279; 280-419; ≥420 g/week.<br>Women: <70; 70-139; ≥140 g/week.<br>**As a continuous variable:** per 280 g/week higher intake.
Drinking frequency (in the past year)	*During the past 12 months, on how many days did you drink alcohol in a typical week?*<br>Daily: 6-7 days/week (i.e., "daily or almost every day").<br>Non-daily: "1-2 days/week" or "3-5 days/week".
Types of alcohol (on a typical drinking day)[a]	Derived from "*On days when you drink, how much alcohol do you usually drink in a day?*", for which participants had to choose one beverage type to report the amount usually drunk on a typical drinking day.<br>Spirits: "Strong spirit (≥40% alcohol)" or "weak spirit (<40% alcohol)".<br>Non-spirits: "Rice wine", "grape wine", or "beer".
Heavy episodic drinking (on a typical drinking day)[a]	Derived based on the amount of alcohol consumed on a typical drinking day.<br>Men: Consumption of >60 g (i.e., 7.5 UK units or 4.3 US standard drinks) of alcohol on a typical drinking day.<br>Women: Consumption of >40 g (i.e., 5 UK units or 2.9 US standard drinks) of alcohol on a typical drinking day.[11]
Drinking with/outside of meals (on a typical drinking day)	*On a typical day when you drink alcohol, when do you usually take the drink?*<br>With meals: "Usually drank with meals".<br>Outside of meals: "Usually drank between or after meals" or "no regular patterns".
Duration of regular drinking	Number of years of regular drinking calculated by the difference between baseline age (years) and age started regular drinking (years).
Flushing response	*After drinking alcohol, do you usually experience hot flushes or dizziness?*<br>No: "Yes, but only after drinking a large amount of alcohol" or "No".<br>Yes: "Yes, soon after the first mouthful" or "Yes, after drinking a small amount of alcohol".

[a]Data was available on a typical drinking day, on special occasions, and the last time the participants drank. For this study, alcohol data reported on a typical drinking day was used in the analyses to reflect the usual drinking habits of the participants.

Table S2. Estimated regression dilution ratio of alcohol consumption in men and women

Baseline-defined groups	N	Mean consumption, g/week: Baseline	1st resurvey	2nd resurvey	Usual	Regression dilution ratio[a]: MacMahon-Peto method	Pearson's correlation	Spearman's correlation	Rosner's regression
**Men**									
Abstainers & ex-regular drinkers	1206	0.0	8.5	13.2	10.9				
Occasional drinkers	2400	5.0	22.5	39.6	31.0				
Current regular drinkers									
<140 g/week	672	77.4	96.2	119.1	107.6				
140-279 g/week	464	223.5	183.5	243.7	213.6				
280-419 g/week	322	367.4	292.1	301.0	296.5				
420+ g/week	364	685.6	440.7	437.1	438.9				
Regression dilution ratio[a]						0.54	0.52	0.55	0.55
**Women**									
Abstainers & ex-regular drinkers	5732	0.0	0.9	0.8	0.9				
Occasional drinkers	3012	5.0	4.4	3.7	4.0				
Current regular drinkers									
<70 g/week	79	33.8	29.9	19.9	24.9				
70-139 g/week	49	115.1	90.8	56.8	73.8				
140+ g/week	49	324.9	215.5	181.5	198.5				
Regression dilution ratio[a]						0.60	0.62	0.53	0.71

HBsAg, hepatitis B surface antigen.
Participants with unclear or missing HBsAg test result, or with self-reported prior cancer, liver cirrhosis, or chronic hepatitis were excluded.
Usual consumption is the average alcohol intake of the two resurveys.
[a]Regression dilution ratio is estimated among baseline current regular drinkers using baseline and usual alcohol intakes. The regression dilution ratios calculated using the MacMahon-Peto method based on baseline survey and first resurvey are 0.57 for men and 0.64 for women, and those based on baseline and second resurvey are 0.52 for men and 0.56 for women.

Table S3. Baseline drinking characteristics of participants by level of alcohol consumption, in male current regular drinkers

	All current regular	Level of alcohol consumption			
		<140 g/week	140-279 g/week	280-419 g/week	≥420 g/week
Number of participants	66977	24171	18182	12306	12318
**Alcohol drinking characteristics**					
Mean alcohol consumption, g/week (SD)	283.9 (244.0)	80.4 (38.8)	221.2 (44.9)	372.9 (47.1)	682.4 (237.2)
Daily drinking, %	61.7	31.2	64.9	81.9	94.6
Heavy episodic drinking on typical day, %	37.1	10.0	14.7	63.4	100.0
Drinking spirits on typical day, %	69.6	53.7	71.1	82.4	87.3
Drinking with meals, %	85.7	86.1	86.3	85.7	83.0
Flushing response, %	17.8	26.5	17.4	14.4	9.2
Mean years of regular drinking, year (SD)	22.9 (12.4)	21.0 (12.7)	23.0 (12.3)	24.2 (11.7)	25.8 (11.4)
Mean age started regular drinking, year (SD)	28.7 (11.0)	30.5 (12.2)	28.7 (10.5)	27.3 (9.7)	25.7 (8.9)

SD, standard deviation; HBsAg, hepatitis B surface antigen.
Participants with unclear or missing HBsAg test result, or with self-reported prior cancer, liver cirrhosis, or chronic hepatitis were excluded.
Prevalences and means are directly standardised to the age and study area structure of male current regular drinkers in the study population.

Table S4. Baseline characteristics of participants by alcohol drinking categories, in women

	Overall	Abstainers	Ex-regular drinkers	Occasional drinkers	Current regular drinkers			
					Current regular	<70 g/week	70-139 g/week	≥140 g/week
Number of participants	291604	185024	2412	98272	5896	3094	1494	1308
**Socio-demographic characteristics**								
Mean age, years (SD)	51.4 (10.5)	52.7 (10.7)	55.4 (9.4)	49.3 (9.9)	52.8 (10.3)	52.9 (10.7)	52.9 (10.1)	51.6 (9.5)
Urban area, %	45.0	43.1	31.6	48.3	48.0	62.5	37.3	27.2
Educational attainment >6 years, %	43.6	41.4	46.4	49.4	48.7	50.6	45.0	47.9
Income >20000 yuan/year, %	40.6	37.9	45.7	44.3	46.9	49.5	44.3	46.4
Married, %	89.0	88.8	84.0	89.3	87.6	88.0	87.7	85.7
**Lifestyle factors**								
Regular smoking, %	2.3	1.8	5.4	2.8	7.8	5.5	8.1	15.8
Daily fresh fruit consumption, %	31.6	29.8	42.3	36.8	38.9	42.4	34.0	32.6
Physical activity, mean MET-h/d (SD)	20.4 (12.7)	20.1 (13.3)	20.6 (11.0)	20.5 (11.6)	20.4 (11.5)	20.2 (11.4)	20.6 (11.7)	19.4 (11.2)
Daily tea drinking, %	16.0	14.9	26.0	19.0	30.9	29.4	34.4	32.9
**Physical measurements, mean (SD)**								
SBP, mmHg	129.9 (22.0)	130.9 (22.5)	130.8 (23.1)	128.0 (20.5)	127.9 (21.4)	127.7 (20.9)	128.1 (21.5)	130.9 (21.8)
DBP, mmHg	76.8 (10.9)	77.1 (11.0)	77.6 (11.2)	76.1 (10.6)	76.5 (10.9)	76.2 (10.7)	77.1 (10.9)	77.6 (11.1)
BMI, kg/m^2	23.8 (3.5)	23.9 (3.5)	24.2 (3.5)	23.8 (3.4)	23.7 (3.4)	23.7 (3.4)	23.8 (3.3)	24.3 (3.4)
**Health and medical history, %[a]**								
Poor health	11.1	12.3	21.1	9.4	7.8	7.5	7.9	8.4
Any chronic disease[b]	21.0	21.9	31.0	19.8	18.5	19.6	17.9	16.7
Coronary heart diseases	3.2	3.5	5.6	2.7	2.5	2.5	2.7	1.6
Stroke or transient ischaemic attack	1.3	1.6	2.6	1.0	0.7	0.7	0.6	1.1
Prevalent diabetes	6.2	7.1	8.4	5.0	3.5	3.7	2.9	4.3
Family history of cancer	16.3	15.6	21.4	17.5	18.6	18.7	16.7	18.9
Family history of diabetes	7.2	6.6	10.1	8.2	8.4	7.7	10.1	7.8
HBsAg test positive	2.6	2.7	2.1	2.4	2.3	2.3	4.1	1.8

SD, standard deviation; MET-h/d, metabolic equivalents of task per hours per day; SBP, systolic blood pressure; DBP, diastolic blood pressure; BMI, body mass index; HBsAg, hepatitis B surface antigen.
Participants with unclear or missing HBsAg test result, or with self-reported prior cancer, liver cirrhosis, or chronic hepatitis were excluded.
Prevalences and means are directly standardised to the age and study area structure of the female study population as appropriate.
[a]All self-reported except for prevalent diabetes which included both self-reported and screen-detected diabetes.
[b]Chronic diseases included self-reported history of coronary heart disease, stroke, transient ischaemic attack, diabetes, tuberculosis, rheumatoid arthritis, peptic ulcer, emphysema/chronic bronchitis, gallstone/gallbladder disease, and kidney disease.

Table S5. Baseline drinking characteristics of participants by level of alcohol consumption, in female current regular drinkers

	All current regular	Level of alcohol consumption		
		<70 g/week	70-139 g/week	≥140 g/week
Number of participants	5896	3094	1494	1308
**Alcohol drinking characteristics**				
Mean alcohol consumption, g/week (SD)	112.5 (123.5)	35.6 (19.2)	110.1 (23.8)	276.9 (134.9)
Daily drinking, %	44.6	22.8	59.3	69.5
Heavy episodic drinking on typical day, %	25.9	9.5	10.1	79.7
Drinking spirits on typical day, %	61.0	55.8	65.7	81.3
Drinking with meals, %	85.8	86.1	84.9	87.6
Flushing response, %	23.5	26.3	22.6	19.5
Mean years of regular drinking, year (SD)	15.2 (12.8)	13.5 (11.7)	16.1 (12.6)	19.1 (12.9)
Mean age started regular drinking, year (SD)	37.8 (13.5)	39.5 (13.6)	37.1 (13.1)	33.9 (11.8)

SD, standard deviation; HBsAg, hepatitis B surface antigen.
Participants with unclear or missing HBsAg test result, or with self-reported prior cancer, liver cirrhosis, or chronic hepatitis were excluded.
Prevalences and means are directly standardised to the age and study area structure of female regular drinkers in the study population.

Table S6. Adjusted HRs for total and major chronic liver diseases associated with drinking status, in women

		Abstainers		Ex-regular drinkers		Occasional drinkers		Current regular drinkers		
	**All N**	**N**	**HR (95% CI)**	**N**	**HR (95% CI)**	**N**	**HR (95% CI)**	**N**	**HR (95% CI)**	***P* value[a]**
Liver cancer	939	679	1.00 (0.90-1.11)	13	1.04 (0.59-1.81)	227	0.78 (0.68-0.90)	20	0.84 (0.54-1.31)	0.452
Liver cirrhosis	942	664	1.00 (0.90-1.11)	16	1.31 (0.78-2.19)	242	0.84 (0.73-0.96)	20	0.86 (0.55-1.35)	0.519
Alcoholic liver disease	21	16	1.00 (0.56-1.79)	1	4.19 (0.58-30.24)	2	0.55 (0.14-2.26)	2	6.22 (1.45-26.74)	0.023
Non-alcoholic fatty liver disease	822	492	1.00 (0.89-1.12)	18	0.78 (0.49-1.24)	265	0.98 (0.87-1.11)	47	1.13 (0.84-1.52)	0.440
Chronic viral hepatitis	689	419	1.00 (0.88-1.13)	10	1.64 (0.87-3.10)	246	1.11 (0.97-1.27)	14	1.00 (0.58-1.71)	0.996
Total liver disease	4498	3044	1.00 (0.95-1.05)	72	1.00 (0.79-1.26)	1259	0.91 (0.86-0.97)	123	0.86 (0.72-1.03)	0.123

HR, hazard ratio; CI, confidence interval; HBsAg, hepatitis B surface antigen.
Participants with unclear or missing HBsAg test result, or with self-reported prior cancer, liver cirrhosis, or chronic hepatitis were excluded.
Cox models are stratified by age-at-risk, study area, and HBsAg, and adjusted for education, household income, smoking, body mass index, and physical activity.

Figure S1. Spline curves of adjusted HRs for total and major chronic liver diseases associated with alcohol intake over the range of 0-1500 g/week, in male current regular drinkers

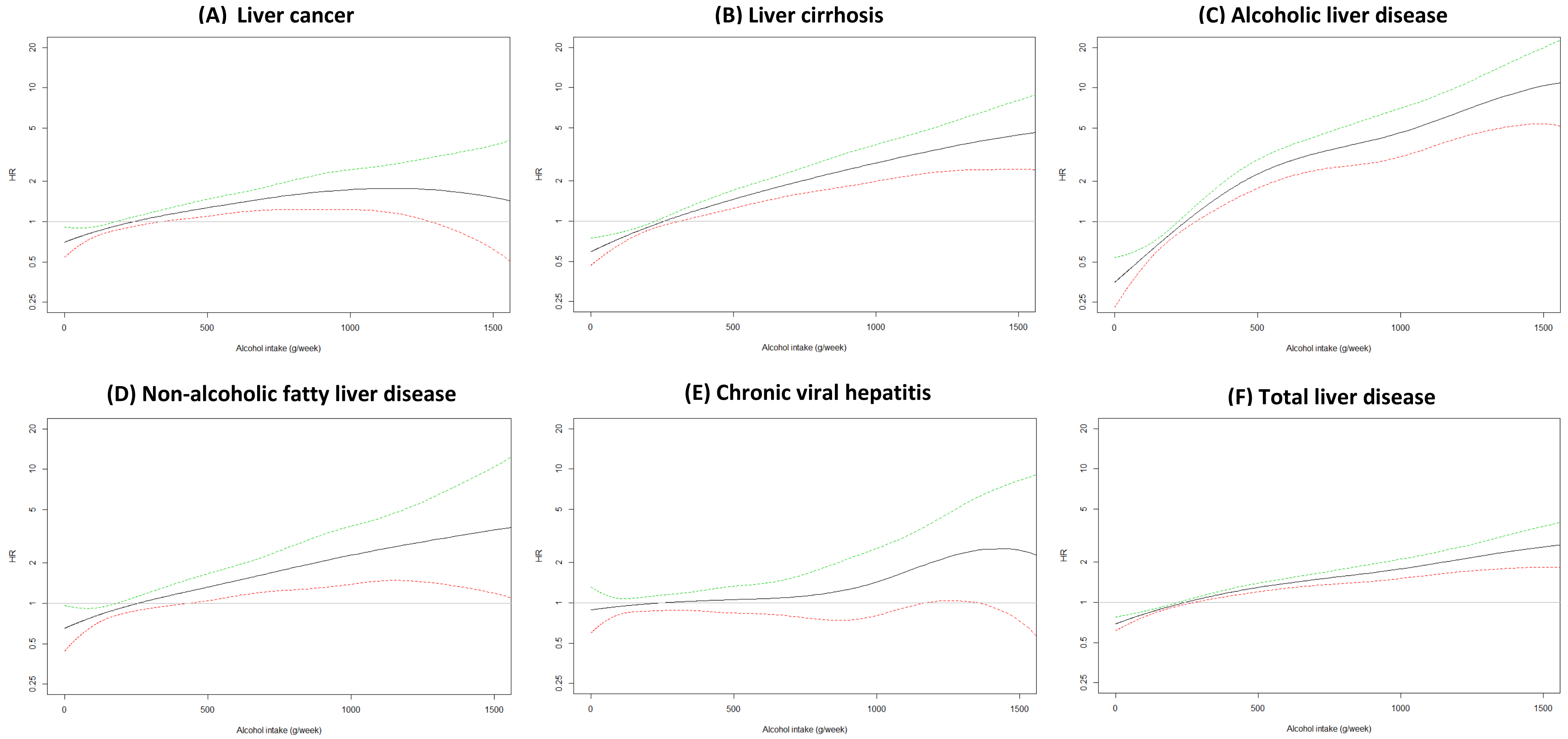

Cox models are stratified by age-at-risk, study area, and HBsAg, and adjusted for education, household income, smoking, physical activity, and body mass index.

Figure S2. Association of alcohol consumption with total liver disease, in female current regular drinkers

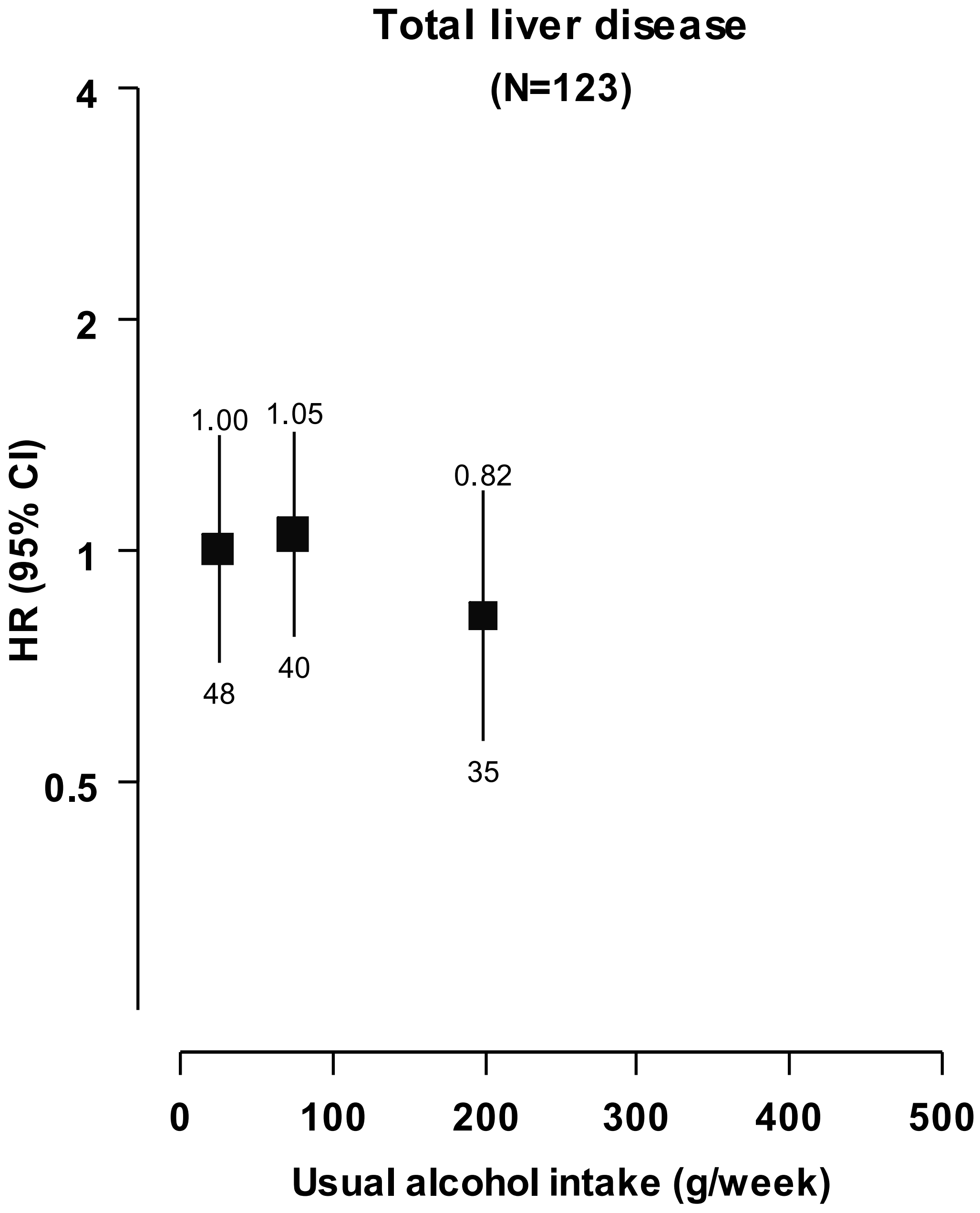

Cox models are stratified by age-at-risk, study area, and HBsAg, and adjusted for education, household income, smoking, physical activity, and body mass index. Each solid square represents an HR. The size of each box is inversely proportional to the "floated" variance of the log hazard in each group and the error bars indicate the group-specific 95% CIs. Group-specific 95% CIs are plotted using floating standard errors to allow for comparison between any two categories. The numbers above the error bars are point estimates for HRs, and the numbers below are number of events. Usual alcohol intake is calculated by the average of the self-reported alcohol intake at the two resurveys in each group. HR per 280 g/week increment in usual alcohol intake = 1.34 (95% CI 0.73-2.46). HR, hazard ratio; CI, confidence interval; HBsAg, hepatitis B surface antigen.

Table S7. Adjusted HRs for total and major chronic liver diseases associated with amount of alcohol consumption, in female current regular drinkers

	All current regular	<70 g/week		≥70 g/week		
	N	N	HR (95% CI)	N	HR (95% CI)	*P* value
Liver cancer	20	8	1.00 (Reference)	12	1.10 (0.35-3.47)	0.869
Liver cirrhosis	20	11	1.00 (Reference)	9	0.60 (0.21-1.67)	0.326
Non-alcoholic fatty liver disease	47	15	1.00 (Reference)	32	1.08 (0.54-2.14)	0.835
Total liver disease	123	48	1.00 (Reference)	75	0.94 (0.63-1.42)	0.776

HR, hazard ratio; CI, confidence interval; HBsAg, hepatitis B surface antigen.
Participants with unclear or missing HBsAg test result, or with self-reported prior cancer, liver cirrhosis, or chronic hepatitis were excluded.
Cox models are stratified by age-at-risk, study area, and HBsAg, and adjusted for education, household income, smoking, body mass index, and physical activity.
Alcoholic liver disease (n=2) and chronic viral hepatitis (n=14) are not shown due to the extremely small numbers of events.

Figure S3. Adjusted HRs per 280g/week higher usual alcohol intake for liver cancer, liver cirrhosis and non-alcoholic fatty liver disease, by population subgroups in male current regular drinkers

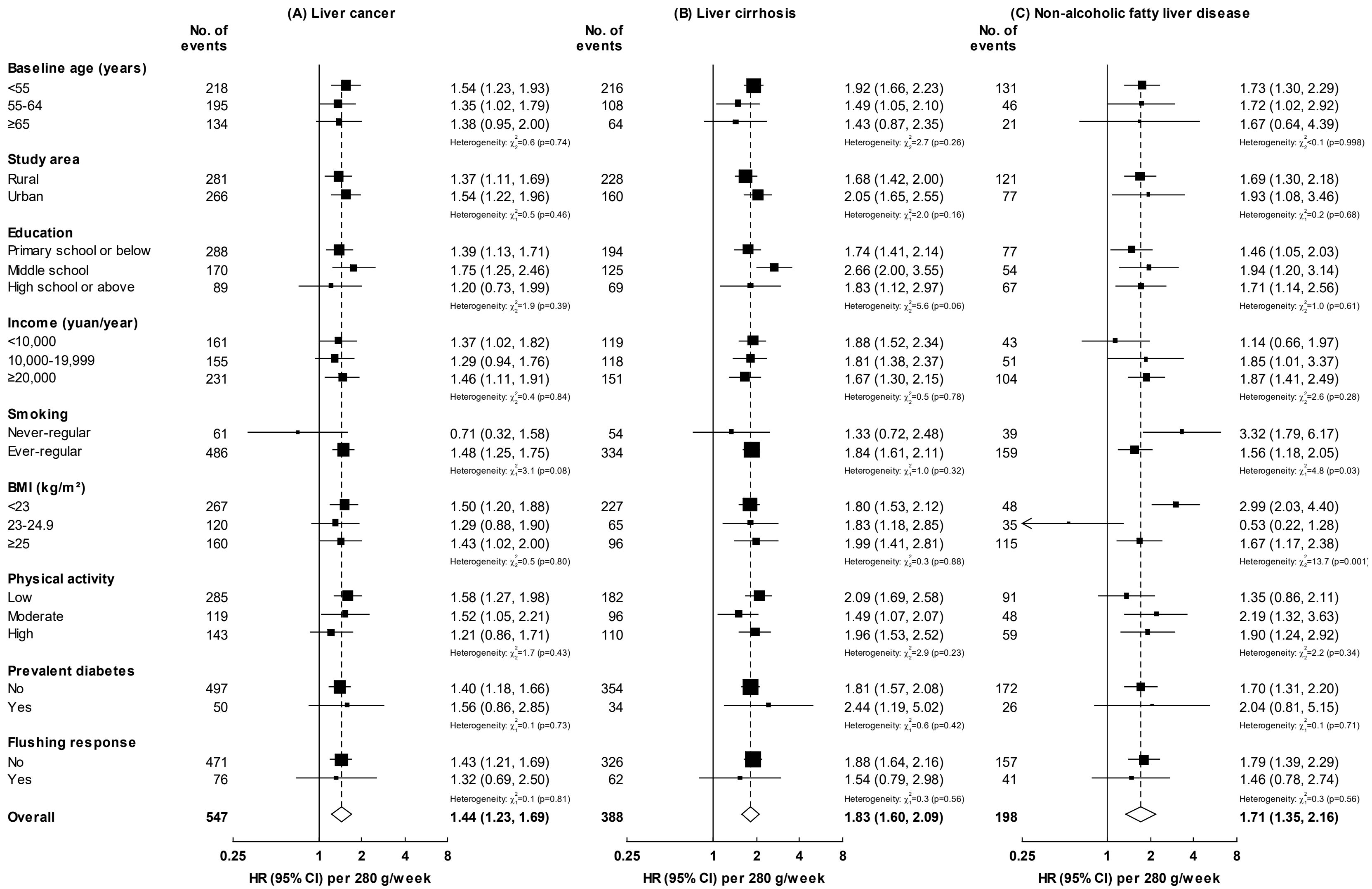

Cox models are stratified by age-at-risk, study area, and HBsAg, and adjusted for education, household income, smoking, physical activity, and body mass index, where appropriate. Each solid square represents an HR. The size of each box is inversely proportional to the variance of the log HR and the error bars indicate the 95% CIs. HR, hazard ratio; CI, confidence interval; HBsAg, hepatitis B surface antigen.

Figure S4. Associations of the joint effects of alcohol consumption and body mass index with total and major chronic liver diseases, in male current regular drinkers

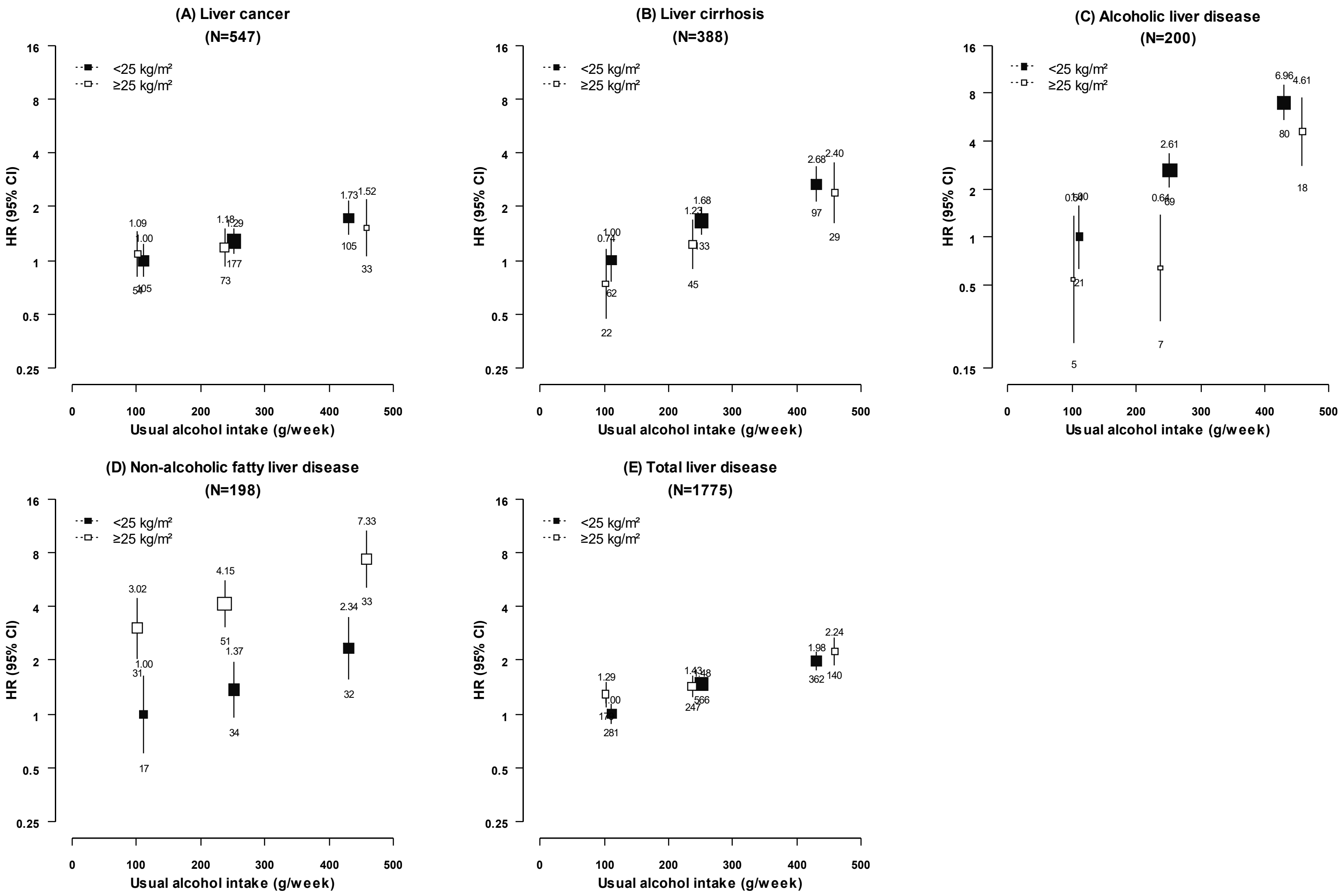

Conventions are as in Figure S2.

Figure S5. Associations of the joint effects of alcohol consumption and HBsAg seropositivity with total and major chronic liver diseases, in male current regular drinkers

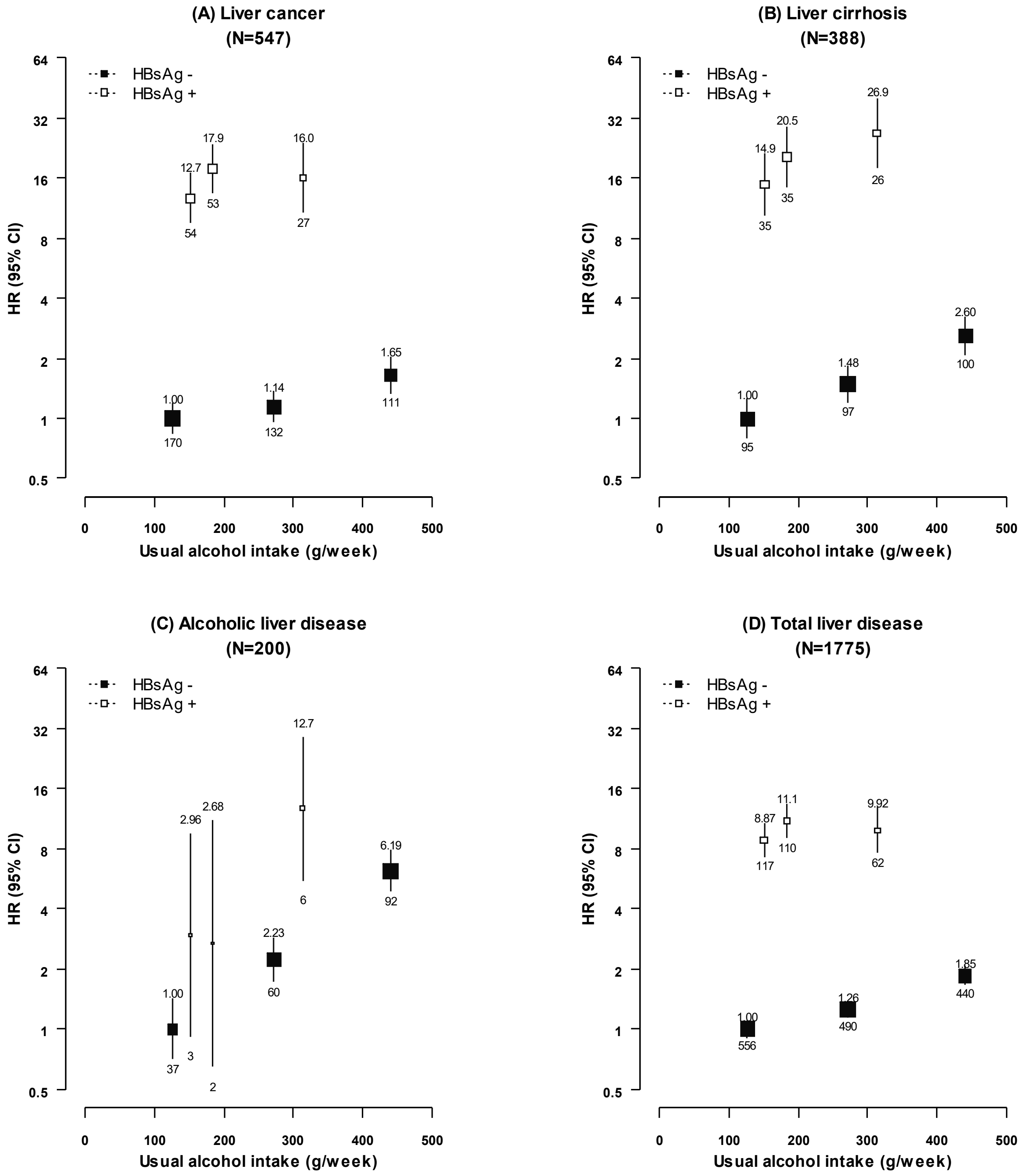

HBsAg (-) included negative, unclear, or missing test results. Non-alcoholic fatty liver disease is not shown here due to low number of events in HBsAg positive participants (n=4). Conventions are as in Figure S2.

Figure S6. Adjusted HRs per 280 g/week higher usual alcohol intake for total and major chronic liver diseases, by HBsAg seroprevalence in male current regular drinkers

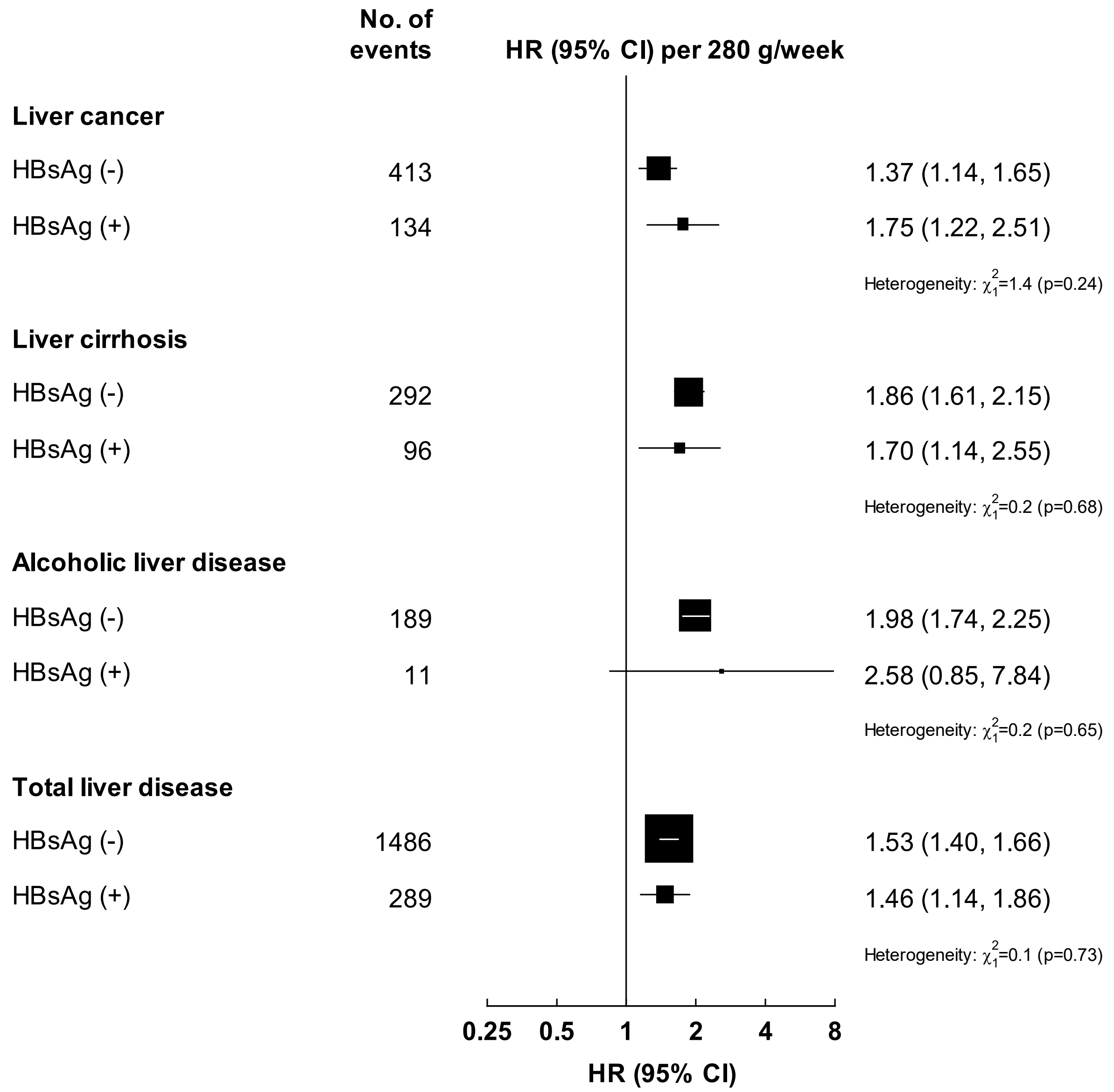

Non-alcoholic fatty liver disease is not shown here due to low number of events in HBsAg positive participants (n=4). Conventions are as in Figure S3.

Table S8. Adjusted HRs for total and major chronic liver diseases associated with daily drinking, by weekly alcohol intake level in male current regular drinkers

			1-5 days/week		6-7 days/week				
					Full model[a]			Further adjusted for total alcohol intake	
		All current regular N	N	HR (95% CI)	N	HR (95% CI)	*P* value	HR (95% CI)	*P* value
Liver cancer	<280 g/week	288	118	1.00 (Reference)	170	1.22 (0.94-1.57)	0.136	1.19 (0.87-1.62)	0.270
	≥280 g/week	259	14	1.00 (Reference)	245	1.19 (0.67-2.14)	0.555	1.13 (0.63-2.03)	0.690
Liver cirrhosis	<280 g/week	190	79	1.00 (Reference)	111	1.39 (1.02-1.91)	0.040	1.01 (0.69-1.49)	0.944
	≥280 g/week	198	13	1.00 (Reference)	185	1.49 (0.78-2.85)	0.225	1.24 (0.64-2.38)	0.524
Alcoholic liver disease	<280 g/week	67	24	1.00 (Reference)	43	1.85 (1.08-3.16)	0.025	1.30 (0.68-2.48)	0.428
	≥280 g/week	133	6	1.00 (Reference)	127	2.04 (0.87-4.76)	0.099	1.63 (0.69-3.83)	0.264
Non-alcoholic fatty liver disease	<280 g/week	97	63	1.00 (Reference)	34	0.68 (0.44-1.07)	0.097	0.55 (0.32-0.93)	0.027
	≥280 g/week	101	7	1.00 (Reference)	94	1.69 (0.74-3.90)	0.216	1.47 (0.63-3.43)	0.369
Chronic viral hepatitis	<280 g/week	129	67	1.00 (Reference)	62	1.18 (0.80-1.73)	0.408	1.35 (0.85-2.16)	0.201
	≥280 g/week	97	15	1.00 (Reference)	82	0.65 (0.35-1.24)	0.195	0.61 (0.32-1.18)	0.145
Total liver disease	<280 g/week	905	413	1.00 (Reference)	492	1.14 (0.99-1.32)	0.069	1.03 (0.86-1.22)	0.772
	≥280 g/week	870	52	1.00 (Reference)	818	1.39 (1.03-1.89)	0.033	1.27 (0.93-1.72)	0.133

HR, hazard ratio; CI, confidence interval; HBsAg, hepatitis B surface antigen.
Participants with unclear or missing HBsAg test result, or with self-reported prior cancer, liver cirrhosis, or chronic hepatitis were excluded.
[a]Cox models are stratified by age-at-risk, study area, and HBsAg, and adjusted for education, household income, smoking, body mass index, and physical activity.

Table S9. Adjusted HRs for total and major chronic liver diseases associated with heavy episodic drinking, by weekly alcohol intake level in male current regular drinkers

			≤60 g/session		>60 g/session (i.e., HED)				
					Full model[a]			Further adjusted for total alcohol intake	
		All current regular N	N	HR (95% CI)	N	HR (95% CI)	*P* value	HR (95% CI)	*P* value
Liver cancer	<280 g/week	288	261	1.00 (Reference)	27	1.06 (0.69-1.62)	0.806	1.04 (0.67-1.60)	0.870
	≥280 g/week	259	56	1.00 (Reference)	203	1.05 (0.74-1.48)	0.790	0.93 (0.63-1.36)	0.701
Liver cirrhosis	<280 g/week	190	168	1.00 (Reference)	22	1.13 (0.70-1.83)	0.609	1.07 (0.66-1.72)	0.794
	≥280 g/week	198	24	1.00 (Reference)	174	1.57 (0.95-2.59)	0.077	1.12 (0.67-1.87)	0.673
Alcoholic liver disease	<280 g/week	67	61	1.00 (Reference)	6	0.78 (0.32-1.91)	0.592	0.72 (0.30-1.76)	0.473
	≥280 g/week	133	11	1.00 (Reference)	122	1.59 (0.80-3.15)	0.188	1.15 (0.57-2.31)	0.692
Non-alcoholic fatty liver disease	<280 g/week	97	80	1.00 (Reference)	17	1.18 (0.67-2.07)	0.563	1.17 (0.66-2.07)	0.588
	≥280 g/week	101	15	1.00 (Reference)	86	0.93 (0.49-1.78)	0.831	0.67 (0.34-1.32)	0.241
Chronic viral hepatitis	<280 g/week	129	105	1.00 (Reference)	24	1.29 (0.79-2.10)	0.316	1.30 (0.79-2.13)	0.294
	≥280 g/week	97	13	1.00 (Reference)	84	1.50 (0.77-2.95)	0.235	1.47 (0.72-3.00)	0.289
Total liver disease	<280 g/week	905	799	1.00 (Reference)	106	1.14 (0.91-1.41)	0.257	1.10 (0.88-1.37)	0.388
	≥280 g/week	870	169	1.00 (Reference)	701	1.05 (0.87-1.28)	0.609	0.86 (0.69-1.05)	0.143

HED, heavy episodic drinking; HR, hazard ratio; CI, confidence interval; HBsAg, hepatitis B surface antigen.
Participants with unclear or missing HBsAg test result, or with self-reported prior cancer, liver cirrhosis, or chronic hepatitis were excluded.
[a]Cox models are stratified by age-at-risk, study area, and HBsAg, and adjusted for education, household income, smoking, body mass index, and physical activity.

Figure S7. Associations of the joint effects of alcohol consumption and mealtime drinking habits with total and major chronic liver diseases, in male current regular drinkers

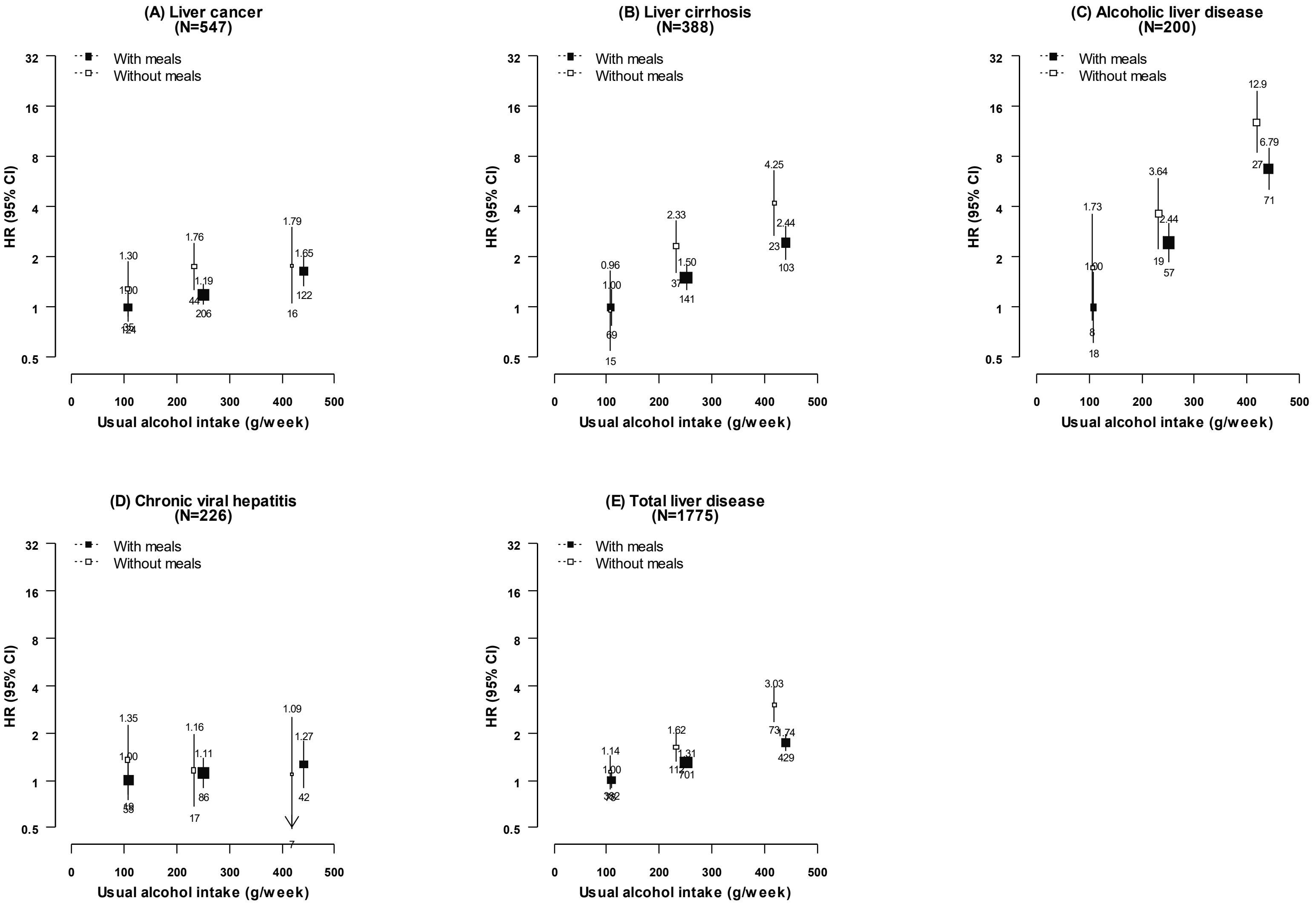

Non-alcoholic fatty liver disease is not shown here due to the low number of events among men usually drinking without meals (n=12). Conventions are as in Figure S2.

Figure S8. Adjusted HRs per 280 g/week higher usual alcohol intake for total and major chronic liver diseases, by beverage type in male current regular drinkers

	No. of events	HR (95% CI) per 280 g/week
**Liver cancer**		
Beer	61	2.25 (1.05, 4.82)
Rice wine or grape wine	75	1.73 (0.92, 3.23)
Spirits	411	1.34 (1.11, 1.62)
		Heterogeneity: χ^2_2=2.2 (p=0.33)
**Liver cirrhosis**		
Beer	46	1.97 (0.82, 4.76)
Rice wine or grape wine	51	2.55 (1.18, 5.51)
Spirits	291	1.84 (1.59, 2.13)
		Heterogeneity: χ^2_2=0.7 (p=0.70)
**Alcoholic liver disease**		
Beer	14	4.41 (1.29, 15.07)
Rice wine or grape wine	15	2.13 (0.54, 8.36)
Spirits	171	2.00 (1.75, 2.29)
		Heterogeneity: χ^2_2=1.6 (p=0.44)
**Non-alcoholic fatty liver disease**		
Beer	41	2.75 (1.11, 6.79)
Rice wine or grape wine	12	1.93 (0.40, 9.32)
Spirits	145	1.63 (1.24, 2.14)
		Heterogeneity: χ^2_2=1.3 (p=0.53)
**Chronic viral hepatitis**		
Beer	45	0.88 (0.27, 2.80)
Rice wine or grape wine	18	2.96 (0.66, 13.21)
Spirits	163	1.28 (0.95, 1.72)
		Heterogeneity: χ^2_2=1.7 (p=0.43)
**Total liver disease**		
Beer	233	1.73 (1.12, 2.67)
Rice wine or grape wine	220	1.54 (1.06, 2.25)
Spirits	1322	1.50 (1.37, 1.64)
		Heterogeneity: χ^2_2=0.4 (p=0.81)

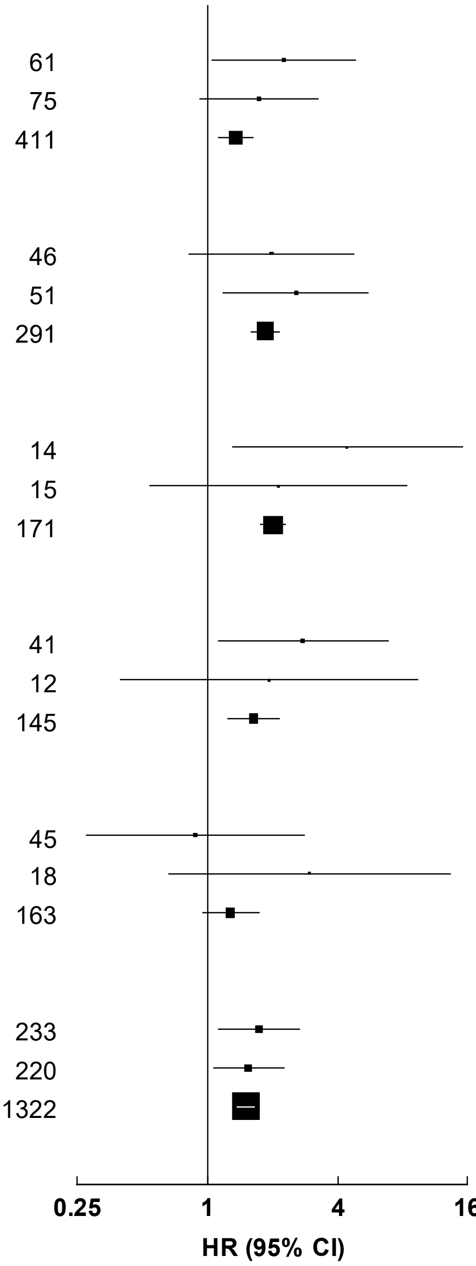

Conventions are as in Figure S3.

Table S10. Adjusted HRs for total and major chronic liver diseases associated with the flushing response, in male current regular drinkers

		No flushing response		Flushing response				
					Full model[a]		+ Total alcohol intake	
	All current regular N	N	HR (95% CI)	N	HR (95% CI)	*P* value	HR (95% CI)	*P* value
Liver cancer	547	471	1.00 (Reference)	76	0.78 (0.61-1.00)	0.054	0.84 (0.66-1.08)	0.178
Liver cirrhosis	388	326	1.00 (Reference)	62	0.85 (0.64-1.12)	0.255	0.98 (0.74-1.30)	0.916
Alcoholic liver disease	200	177	1.00 (Reference)	23	0.55 (0.35-0.85)	0.007	0.66 (0.43-1.03)	0.069
Non-alcoholic fatty liver disease	198	157	1.00 (Reference)	41	1.06 (0.75-1.51)	0.744	1.19 (0.84-1.70)	0.335
Chronic viral hepatitis	226	180	1.00 (Reference)	46	1.21 (0.87-1.69)	0.261	1.27 (0.90-1.77)	0.170
Total liver disease	1775	1479	1.00 (Reference)	296	0.92 (0.81-1.05)	0.222	1.01 (0.89-1.15)	0.867

HR, hazard ratio; CI, confidence interval; HBsAg, hepatitis B surface antigen.
Participants with unclear or missing HBsAg test result, or with self-reported prior cancer, liver cirrhosis, or chronic hepatitis were excluded.
[a]Cox models are stratified by age-at-risk, study area, and HBsAg, and adjusted for education, household income, smoking, body mass index, and physical activity.

Table S11. Adjusted HRs per 280 g/week higher usual alcohol intake for total and major chronic liver diseases, in male current regular drinkers – in sequentially adjusted models

		Basic model			Gradual adjustment for major confounding factors				Further adjustment			
		Age, study area	+ Education	+ Income	+ HBsAg	+ Smoking	+ BMI	+ Physical activity	+ Family history of cancer	+ Prevalent diabetes	+ Systolic blood pressure	+ Family history of diabetes
	N	HR (95% CI)	HR (95% CI)	HR (95% CI)	HR (95% CI)	HR (95% CI)	HR (95% CI)	HR (95% CI)	HR (95% CI)	HR (95% CI)	HR (95% CI)	HR (95% CI)
Liver cancer	547	1.51 (1.30-1.76)	1.51 (1.30-1.75)	1.51 (1.30-1.75)	1.49 (1.27-1.74)	1.44 (1.23-1.69)	1.44 (1.23-1.69)	1.44 (1.23-1.69)	1.44 (1.23-1.69)	1.44 (1.22-1.69)	1.44 (1.22-1.69)	1.44 (1.22-1.69)
Liver cirrhosis	388	1.84 (1.62-2.09)	1.82 (1.60-2.07)	1.82 (1.60-2.06)	1.81 (1.59-2.07)	1.83 (1.60-2.09)	1.83 (1.60-2.09)	1.83 (1.60-2.09)	1.83 (1.60-2.10)	1.83 (1.60-2.10)	1.81 (1.58-2.08)	1.81 (1.58-2.08)
Alcoholic liver disease	200	1.98 (1.77-2.21)	1.96 (1.75-2.19)	2.00 (1.78-2.24)	2.01 (1.79-2.25)	1.96 (1.73-2.21)	1.99 (1.76-2.26)	2.01 (1.77-2.28)	2.01 (1.77-2.28)	2.01 (1.77-2.28)	1.96 (1.73-2.23)	1.96 (1.72-2.23)
Non-alcoholic fatty liver disease	198	1.67 (1.33-2.10)	1.73 (1.37-2.18)	1.73 (1.38-2.18)	1.73 (1.37-2.17)	1.71 (1.35-2.16)	1.71 (1.35-2.16)	1.71 (1.35-2.16)	1.71 (1.35-2.16)	1.70 (1.34-2.16)	1.69 (1.33-2.14)	1.69 (1.33-2.15)
Chronic viral hepatitis	226	1.29 (1.00-1.66)	1.27 (0.98-1.64)	1.27 (0.98-1.64)	1.23 (0.95-1.60)	1.24 (0.95-1.61)	1.24 (0.95-1.62)	1.23 (0.94-1.60)	1.23 (0.94-1.61)	1.22 (0.94-1.60)	1.23 (0.94-1.61)	1.24 (0.95-1.62)
Total liver disease	1775	1.55 (1.44-1.67)	1.54 (1.43-1.66)	1.54 (1.43-1.66)	1.53 (1.42-1.65)	1.52 (1.40-1.64)	1.52 (1.40-1.65)	1.52 (1.40-1.64)	1.52 (1.40-1.64)	1.51 (1.40-1.64)	1.51 (1.39-1.63)	1.51 (1.39-1.63)

HBsAg, hepatitis B surface antigen; HR, hazard ratio; CI, confidence interval.
Participants with unclear or missing HBsAg test result, or with self-reported prior self-reported cancer, liver cirrhosis, or chronic hepatitis were excluded.

Table S12. Adjusted HRs for total and major chronic liver diseases associated with daily drinking, in male current regular drinkers - in sequentially adjusted models

		1-5 days/week		6-7 days/week								
					Full model[a]		+ Total alcohol intake		+ Mealtime habit		+ Beverage type	
	All current regular N	N	HR (95% CI)	N	HR (95% CI)	*P* value	HR (95% CI)	*P* value	HR (95% CI)	*P* value	HR (95% CI)	*P* value
Liver cancer	547	132	1.00 (Reference)	415	1.41 (1.14-1.74)	0.002	1.22 (0.97-1.54)	0.088	1.23 (0.97-1.54)	0.084	1.23 (0.98-1.55)	0.078
Liver cirrhosis	388	92	1.00 (Reference)	296	1.66 (1.29-2.14)	<0.001	1.24 (0.94-1.62)	0.122	1.24 (0.95-1.63)	0.112	1.22 (0.93-1.61)	0.143
Alcoholic liver disease	200	30	1.00 (Reference)	170	3.15 (2.10-4.72)	<0.001	2.15 (1.41-3.28)	<0.001	2.14 (1.41-3.26)	<0.001	2.06 (1.35-3.14)	<0.001
Non-alcoholic fatty liver disease	198	70	1.00 (Reference)	128	1.13 (0.82-1.56)	0.466	0.83 (0.58-1.19)	0.310	0.83 (0.58-1.19)	0.312	0.83 (0.57-1.19)	0.300
Chronic viral hepatitis	226	82	1.00 (Reference)	144	1.11 (0.82-1.50)	0.497	1.00 (0.72-1.41)	0.979	1.01 (0.72-1.41)	0.973	1.02 (0.73-1.43)	0.911
Total liver disease	1775	465	1.00 (Reference)	1310	1.41 (1.25-1.58)	<0.001	1.17 (1.03-1.32)	0.015	1.17 (1.03-1.33)	0.014	1.16 (1.02-1.31)	0.023

HR, hazard ratio; CI, confidence interval; HBsAg, hepatitis B surface antigen.
Participants with unclear or missing HBsAg test result, or with self-reported prior cancer, liver cirrhosis, or chronic hepatitis were excluded.
[a]Cox models are stratified by age-at-risk, study area, and HBsAg, and adjusted for education, household income, smoking, body mass index, and physical activity.

Table S13. Adjusted HRs for total and major chronic liver diseases associated with heavy episodic drinking, in male current regular drinkers - in sequentially adjusted models

		≤60 g/session		>60 g/session (i.e., HED)								
					Full model[a]		+ Total alcohol intake		+ Mealtime habit		+ Beverage type	
	All current regular N	N	HR (95% CI)	N	HR (95% CI)	*P* value	HR (95% CI)	*P* value	HR (95% CI)	*P* value	HR (95% CI)	*P* value
Liver cancer	547	317	1.00 (Reference)	230	1.36 (1.13-1.64)	0.001	1.10 (0.86-1.39)	0.457	1.09 (0.86-1.39)	0.476	1.08 (0.85-1.38)	0.517
Liver cirrhosis	388	192	1.00 (Reference)	196	1.64 (1.32-2.04)	<0.001	1.09 (0.84-1.40)	0.530	1.08 (0.84-1.40)	0.534	1.14 (0.88-1.48)	0.334
Alcoholic liver disease	200	72	1.00 (Reference)	128	2.69 (1.98-3.66)	<0.001	1.69 (1.21-2.37)	0.002	1.72 (1.23-2.42)	0.002	1.72 (1.21-2.44)	0.002
Non-alcoholic fatty liver disease	198	95	1.00 (Reference)	103	1.46 (1.08-1.98)	0.013	1.05 (0.73-1.51)	0.791	1.05 (0.73-1.51)	0.797	1.05 (0.72-1.52)	0.806
Chronic viral hepatitis	226	118	1.00 (Reference)	108	1.32 (0.99-1.76)	0.054	1.27 (0.89-1.81)	0.186	1.27 (0.89-1.81)	0.184	1.27 (0.89-1.81)	0.190
Total liver disease	1775	968	1.00 (Reference)	807	1.38 (1.24-1.52)	<0.001	1.04 (0.92-1.18)	0.501	1.05 (0.92-1.19)	0.492	1.05 (0.92-1.19)	0.459

HED, heavy episodic drinking; HR, hazard ratio; CI, confidence interval; HBsAg, hepatitis B surface antigen.
Participants with unclear or missing HBsAg test result, or with self-reported prior cancer, liver cirrhosis, or chronic hepatitis were excluded.
[a]Cox models are stratified by age-at-risk, study area, and HBsAg, and adjusted for education, household income, smoking, body mass index, and physical activity.

Table S14. Adjusted HRs for total and major chronic liver diseases associated with mealtime drinking habits, in male current regular drinkers - in sequentially adjusted models

		With meals		Without meals						
					Full model[a]		+ Total alcohol intake		+ Beverage type	
	All current regular N	N	HR (95% CI)	N	HR (95% CI)	*P* value	HR (95% CI)	*P* value	HR (95% CI)	*P* value
Liver cancer	547	452	1.00 (Reference)	95	1.35 (1.04-1.75)	0.023	1.32 (1.02-1.71)	0.036	1.32 (1.02-1.71)	0.035
Liver cirrhosis	388	313	1.00 (Reference)	75	1.47 (1.10-1.97)	0.010	1.37 (1.02-1.84)	0.035	1.37 (1.02-1.84)	0.036
Alcoholic liver disease	200	146	1.00 (Reference)	54	1.84 (1.28-2.64)	0.001	1.60 (1.10-2.32)	0.014	1.60 (1.10-2.33)	0.013
Non-alcoholic fatty liver disease	198	186	1.00 (Reference)	12	0.84 (0.45-1.55)	0.573	0.81 (0.44-1.49)	0.492	0.81 (0.44-1.49)	0.495
Chronic viral hepatitis	226	183	1.00 (Reference)	43	1.13 (0.76-1.66)	0.550	1.12 (0.76-1.65)	0.578	1.11 (0.75-1.64)	0.591
Total liver disease	1775	1512	1.00 (Reference)	263	1.33 (1.14-1.54)	<0.001	1.29 (1.11-1.50)	0.001	1.29 (1.11-1.50)	0.001

HR, hazard ratio; CI, confidence interval; HBsAg, hepatitis B surface antigen.
Participants with unclear or missing HBsAg test result, or with self-reported prior cancer, liver cirrhosis, or chronic hepatitis were excluded.
[a]Cox models are stratified by age-at-risk, study area, and HBsAg, and adjusted for education, household income, smoking, body mass index, and physical activity.

Table S15. Adjusted HRs for total and major chronic liver diseases associated with beverage type, in male current regular drinkers - in sequentially adjusted models

		Non-spirits		Spirits						
				Full model[a]		+ Total alcohol intake		+ Mealtime habits		
	All current regular N	N	HR (95% CI)	N	HR (95% CI)	*P* value	HR (95% CI)	*P* value	HR (95% CI)	*P* value
Liver cancer	547	136	1.00 (Reference)	411	1.18 (0.94-1.48)	0.152	1.06 (0.84-1.33)	0.645	1.06 (0.84-1.34)	0.625
Liver cirrhosis	388	97	1.00 (Reference)	291	1.02 (0.77-1.34)	0.895	0.82 (0.62-1.09)	0.164	0.82 (0.62-1.09)	0.176
Alcoholic liver disease	200	29	1.00 (Reference)	171	1.73 (1.10-2.70)	0.017	1.32 (0.84-2.09)	0.228	1.34 (0.85-2.12)	0.210
Non-alcoholic fatty liver disease	198	53	1.00 (Reference)	145	1.21 (0.83-1.76)	0.327	1.04 (0.71-1.53)	0.846	1.04 (0.70-1.53)	0.860
Chronic viral hepatitis	226	63	1.00 (Reference)	163	0.99 (0.70-1.41)	0.976	0.93 (0.65-1.33)	0.692	0.93 (0.65-1.34)	0.696
Total liver disease	1775	453	1.00 (Reference)	1322	1.17 (1.03-1.32)	0.015	1.02 (0.90-1.16)	0.743	1.03 (0.90-1.16)	0.702

HR, hazard ratio; CI, confidence interval; HBsAg, hepatitis B surface antigen.
Participants with unclear or missing HBsAg test result, or with self-reported prior cancer, liver cirrhosis, or chronic hepatitis were excluded.
[a]Cox models are stratified by age-at-risk, study area, and HBsAg, and adjusted for education, household income, smoking, body mass index, and physical activity.

Figure S9. Associations of alcohol consumption with total and major chronic liver diseases, in all men

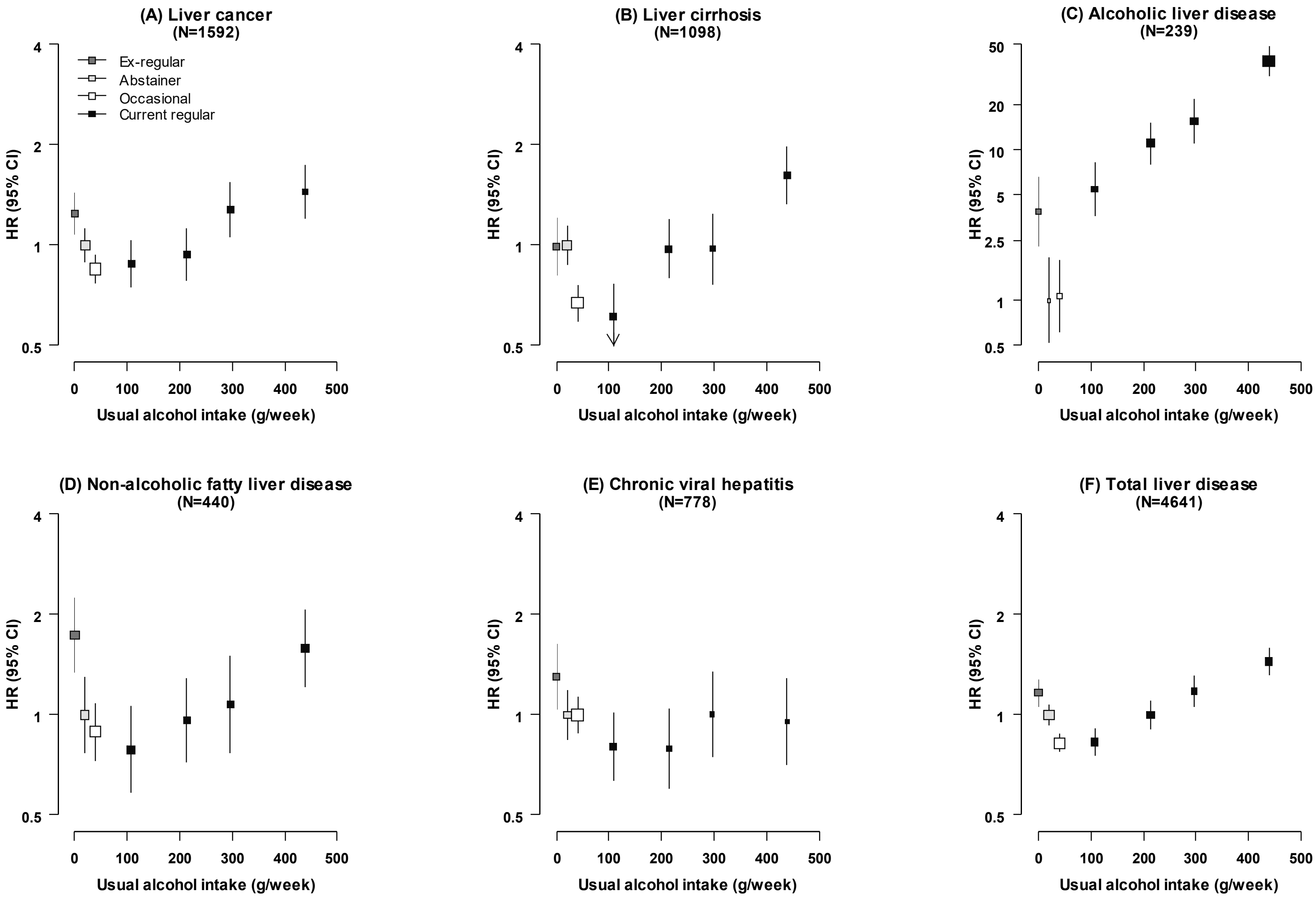

Conventions are as in Figure S2.

Table S16. Adjusted HRs for total and major chronic liver diseases associated with alcohol drinking status, in men after various exclusions of participants

	All	Abstainers		Ex-regular drinkers		Occasional drinkers		Current regular drinkers		
	N	N	HR (95% CI)	N	HR (95% CI)	N	HR (95% CI)	N	HR (95% CI)	*P* value[a]
**Liver cancer**										
Main analysis	1592	365	1.00 (0.90-1.12)	203	1.24 (1.08-1.43)	477	0.86 (0.78-0.94)	547	1.07 (0.98-1.17)	0.364
Excluded first three years of follow-up	1008	215	1.00 (0.87-1.15)	117	1.18 (0.98-1.42)	318	0.91 (0.81-1.02)	358	1.14 (1.02-1.27)	0.172
Excluded poor self-reported health	1409	309	1.00 (0.89-1.13)	159	1.25 (1.06-1.46)	439	0.90 (0.81-0.99)	502	1.09 (1.00-1.20)	0.251
Excluded prior chronic disease[b]	1088	239	1.00 (0.87-1.15)	112	1.23 (1.02-1.49)	337	0.83 (0.74-0.93)	400	1.06 (0.96-1.18)	0.494
Excluded all of above	653	133	1.00 (0.83-1.20)	60	1.22 (0.94-1.57)	215	0.90 (0.78-1.04)	245	1.09 (0.95-1.25)	0.458
**Liver cirrhosis**										
Main analysis	1098	291	1.00 (0.88-1.14)	112	0.97 (0.81-1.18)	307	0.68 (0.60-0.76)	388	0.96 (0.86-1.07)	0.645
Excluded first three years of follow-up	708	170	1.00 (0.85-1.18)	66	1.01 (0.79-1.29)	221	0.81 (0.70-0.92)	251	1.09 (0.95-1.25)	0.431
Excluded poor self-reported health	962	241	1.00 (0.87-1.15)	88	1.03 (0.83-1.27)	288	0.75 (0.66-0.84)	345	0.99 (0.88-1.11)	0.921
Excluded prior chronic disease[b]	752	173	1.00 (0.85-1.18)	63	1.09 (0.84-1.40)	223	0.73 (0.64-0.84)	293	1.09 (0.96-1.24)	0.422
Excluded all of above	463	96	1.00 (0.80-1.24)	34	1.12 (0.79-1.58)	150	0.81 (0.69-0.96)	183	1.21 (1.02-1.42)	0.184
**Alcoholic liver disease**										
Main analysis	239	10	1.00 (0.53-1.87)	15	3.72 (2.24-6.20)	14	1.11 (0.65-1.88)	200	14.03 (12.00-16.41)	<0.001
Excluded first three years of follow-up	173	7	1.00 (0.47-2.12)	9	3.26 (1.69-6.29)	13	1.52 (0.88-2.63)	144	14.63 (12.15-17.62)	<0.001
Excluded poor self-reported health	205	8	1.00 (0.50-2.02)	12	4.08 (2.31-7.22)	12	1.15 (0.65-2.03)	173	14.97 (12.65-17.72)	<0.001
Excluded prior chronic disease[b]	179	6	1.00 (0.45-2.24)	9	4.27 (2.21-8.25)	8	0.99 (0.49-1.99)	156	16.73 (14.02-19.97)	<0.001
Excluded all of above	115	2	1.00 (0.25-4.03)	3	4.42 (1.42-13.76)	7	2.60 (1.23-5.50)	103	33.17 (26.64-41.30)	<0.001
**Non-alcoholic fatty liver disease**										
Main analysis	440	64	1.00 (0.77-1.30)	67	1.73 (1.35-2.22)	111	0.91 (0.75-1.10)	198	1.07 (0.93-1.24)	0.645
Excluded first three years of follow-up	257	18	1.00 (0.62-1.61)	56	2.83 (2.15-3.72)	57	1.02 (0.78-1.34)	126	1.37 (1.14-1.65)	0.228
Excluded poor self-reported health	391	57	1.00 (0.76-1.31)	51	1.69 (1.27-2.24)	106	0.99 (0.81-1.20)	177	1.08 (0.92-1.26)	0.651
Excluded prior chronic disease[b]	282	41	1.00 (0.73-1.38)	39	2.13 (1.53-2.94)	74	0.99 (0.78-1.26)	128	1.15 (0.96-1.38)	0.455
Excluded all of above	137	9	1.00 (0.51-1.96)	25	3.02 (2.00-4.57)	34	1.15 (0.81-1.64)	69	1.36 (1.06-1.75)	0.401
**Chronic viral hepatitis**										
Main analysis	778	162	1.00 (0.85-1.18)	82	1.30 (1.04-1.62)	308	1.00 (0.89-1.13)	226	0.86 (0.75-0.99)	0.196
Excluded first three years of follow-up	494	84	1.00 (0.79-1.26)	46	1.39 (1.04-1.87)	207	1.22 (1.06-1.41)	157	1.16 (0.98-1.37)	0.327
Excluded poor self-reported health	691	137	1.00 (0.83-1.20)	65	1.38 (1.07-1.77)	275	1.04 (0.91-1.17)	214	0.95 (0.82-1.10)	0.678
Excluded prior chronic disease[b]	568	99	1.00 (0.81-1.24)	53	1.65 (1.25-2.17)	250	1.25 (1.09-1.42)	166	0.99 (0.84-1.16)	0.926
Excluded all of above	322	40	1.00 (0.72-1.39)	24	2.10 (1.40-3.16)	149	1.73 (1.46-2.05)	109	1.68 (1.37-2.07)	0.009
**Total liver disease**										
Main analysis	4641	1021	1.00 (0.94-1.07)	513	1.16 (1.06-1.27)	1332	0.83 (0.79-0.88)	1775	1.07 (1.01-1.12)	0.136
Excluded first three years of follow-up	2782	516	1.00 (0.91-1.10)	303	1.21 (1.08-1.36)	849	0.92 (0.86-0.99)	1114	1.21 (1.13-1.29)	0.001
Excluded poor self-reported health	4092	878	1.00 (0.93-1.07)	397	1.16 (1.05-1.28)	1213	0.86 (0.81-0.91)	1604	1.08 (1.03-1.14)	0.080
Excluded prior chronic disease[b]	3265	664	1.00 (0.92-1.09)	298	1.26 (1.12-1.41)	978	0.88 (0.82-0.94)	1325	1.13 (1.07-1.20)	0.016
Excluded all of above	1791	303	1.00 (0.89-1.13)	149	1.28 (1.08-1.50)	570	0.95 (0.87-1.04)	769	1.29 (1.19-1.40)	<0.001

HBsAg, hepatitis B surface antigen; HR, hazard ratio; CI, confidence interval.
Participants with unclear or missing HBsAg test result, or with self-reported prior cancer, liver cirrhosis, or chronic hepatitis were excluded from all analyses.
Cox models are stratified by age-at-risk, study area, and HBsAg, and adjusted for education, household income, smoking, body mass index, and physical activity.
[a]*P* for association comparing current regular drinkers vs. abstainers.
[b]Prior chronic diseases included self-reported prior coronary heart diseases, stroke, transient ischaemic attack, tuberculosis, rheumatoid arthritis, peptic ulcer, emphysema/bronchitis, gallstone/gallbladder disease, kidney disease, and prevalent diabetes (both self-reported and screen-detected).

Table S17. Adjusted HRs for total and major chronic liver diseases associated with amount of alcohol consumption, in male current regular drinkers after various exclusions of participants

		<140 g/week		140-279 g/week		280-419 g/week		≥420 g/week		Per 280 g/week usual intake		
	All current regular N	N	HR (95% CI)	N	HR (95% CI)	N	HR (95% CI)	N	HR (95% CI)	N	HR (95% CI)	*p* trend
**Liver cancer**												
Main analysis	547	159	1.00 (0.84-1.19)	129	1.07 (0.90-1.27)	121	1.45 (1.21-1.73)	138	1.64 (1.37-1.97)	547	1.44 (1.23-1.69)	<0.001
Excluded first three years of follow-up	358	107	1.00 (0.81-1.23)	83	1.04 (0.84-1.29)	76	1.39 (1.11-1.74)	92	1.65 (1.32-2.07)	358	1.49 (1.23-1.81)	<0.001
Excluded poor self-reported health	502	150	1.00 (0.84-1.20)	114	1.01 (0.84-1.21)	109	1.39 (1.15-1.68)	129	1.67 (1.38-2.01)	502	1.48 (1.25-1.75)	<0.001
Excluded prior chronic disease[a]	400	106	1.00 (0.81-1.23)	95	1.11 (0.91-1.36)	95	1.58 (1.29-1.93)	104	1.73 (1.40-2.13)	400	1.48 (1.22-1.80)	<0.001
Excluded all of above	245	70	1.00 (0.77-1.30)	58	1.06 (0.81-1.37)	53	1.40 (1.07-1.84)	64	1.63 (1.24-2.13)	245	1.48 (1.15-1.91)	0.003
**Liver cirrhosis**												
Main analysis	388	84	1.00 (0.80-1.26)	106	1.68 (1.38-2.03)	72	1.66 (1.31-2.09)	126	2.79 (2.30-3.40)	388	1.83 (1.60-2.09)	<0.001
Excluded first three years of follow-up	251	52	1.00 (0.75-1.34)	69	1.78 (1.40-2.25)	46	1.67 (1.25-2.25)	84	3.13 (2.46-3.97)	251	1.83 (1.55-2.17)	<0.001
Excluded poor self-reported health	345	78	1.00 (0.79-1.27)	95	1.65 (1.35-2.02)	65	1.67 (1.31-2.13)	107	2.65 (2.14-3.27)	345	1.84 (1.59-2.14)	<0.001
Excluded prior chronic disease[a]	293	61	1.00 (0.76-1.31)	79	1.70 (1.36-2.13)	59	1.85 (1.43-2.40)	94	2.81 (2.24-3.51)	293	1.82 (1.55-2.14)	<0.001
Excluded all of above	183	40	1.00 (0.72-1.40)	51	1.72 (1.30-2.28)	35	1.80 (1.29-2.52)	57	2.86 (2.15-3.82)	183	1.81 (1.47-2.23)	<0.001
**Alcoholic liver disease**												
Main analysis	200	26	1.00 (0.67-1.49)	41	2.04 (1.50-2.78)	35	2.92 (2.10-4.05)	98	7.24 (5.80-9.04)	200	2.01 (1.77-2.28)	<0.001
Excluded first three years of follow-up	144	19	1.00 (0.63-1.60)	28	1.87 (1.28-2.72)	26	2.81 (1.92-4.12)	71	6.80 (5.24-8.82)	144	2.01 (1.74-2.34)	<0.001
Excluded poor self-reported health	173	22	1.00 (0.65-1.55)	34	1.99 (1.42-2.79)	30	2.98 (2.09-4.24)	87	7.60 (6.00-9.61)	173	2.07 (1.81-2.38)	<0.001
Excluded prior chronic disease[a]	156	21	1.00 (0.64-1.56)	30	1.79 (1.25-2.57)	26	2.49 (1.70-3.64)	79	6.59 (5.16-8.42)	156	2.06 (1.77-2.41)	<0.001
Excluded all of above	103	14	1.00 (0.58-1.72)	19	1.68 (1.07-2.65)	18	2.52 (1.59-3.99)	52	6.44 (4.76-8.71)	103	2.22 (1.79-2.75)	<0.001
**Non-alcoholic fatty liver disease**												
Main analysis	198	48	1.00 (0.73-1.36)	49	1.28 (0.97-1.69)	36	1.49 (1.08-2.06)	65	2.37 (1.79-3.13)	198	1.71 (1.35-2.16)	<0.001
Excluded first three years of follow-up	126	37	1.00 (0.70-1.43)	29	0.93 (0.65-1.33)	19	1.02 (0.65-1.59)	41	1.85 (1.28-2.67)	126	1.78 (1.29-2.45)	<0.001
Excluded poor self-reported health	177	42	1.00 (0.72-1.39)	43	1.32 (0.98-1.78)	34	1.67 (1.20-2.33)	58	2.60 (1.93-3.50)	177	1.75 (1.32-2.31)	<0.001
Excluded prior chronic disease[a]	128	29	1.00 (0.67-1.49)	30	1.23 (0.86-1.76)	25	1.54 (1.04-2.27)	44	2.21 (1.58-3.09)	128	1.50 (1.06-2.11)	0.020
Excluded all of above	69	19	1.00 (0.60-1.66)	14	0.82 (0.49-1.38)	12	1.16 (0.66-2.02)	24	1.77 (1.09-2.87)	69	1.36 (0.80-2.31)	0.251
**Chronic viral hepatitis**												
Main analysis	226	74	1.00 (0.78-1.28)	55	0.94 (0.72-1.22)	48	1.23 (0.92-1.64)	49	1.18 (0.87-1.59)	226	1.23 (0.94-1.60)	0.135
Excluded first three years of follow-up	157	53	1.00 (0.75-1.34)	40	0.94 (0.69-1.28)	33	1.18 (0.83-1.67)	31	1.01 (0.69-1.47)	157	1.03 (0.73-1.47)	0.854
Excluded poor self-reported health	214	70	1.00 (0.77-1.29)	51	0.92 (0.70-1.21)	46	1.28 (0.95-1.71)	47	1.22 (0.89-1.66)	214	1.26 (0.96-1.66)	0.098
Excluded prior chronic disease[a]	166	51	1.00 (0.74-1.35)	44	1.03 (0.76-1.38)	36	1.26 (0.90-1.76)	35	1.10 (0.77-1.56)	166	1.15 (0.82-1.61)	0.411
Excluded all of above	109	38	1.00 (0.71-1.42)	28	0.86 (0.59-1.25)	22	1.10 (0.72-1.69)	21	0.91 (0.58-1.44)	109	0.96 (0.61-1.51)	0.864
**Total liver disease**												
Main analysis	1775	460	1.00 (0.91-1.10)	445	1.24 (1.13-1.36)	368	1.49 (1.34-1.65)	502	1.89 (1.72-2.08)	1775	1.52 (1.40-1.64)	<0.001
Excluded first three years of follow-up	1114	306	1.00 (0.88-1.13)	281	1.17 (1.04-1.32)	212	1.33 (1.16-1.52)	315	1.83 (1.62-2.07)	1114	1.52 (1.37-1.68)	<0.001
Excluded poor self-reported health	1604	424	1.00 (0.90-1.11)	397	1.21 (1.10-1.33)	334	1.49 (1.34-1.66)	449	1.89 (1.71-2.09)	1604	1.55 (1.42-1.69)	<0.001
Excluded prior chronic disease[a]	1325	322	1.00 (0.89-1.13)	328	1.24 (1.11-1.39)	282	1.51 (1.35-1.70)	393	1.94 (1.74-2.17)	1325	1.53 (1.39-1.68)	<0.001
Excluded all of above	769	206	1.00 (0.86-1.16)	194	1.14 (0.99-1.32)	145	1.28 (1.09-1.51)	224	1.80 (1.55-2.08)	769	1.52 (1.34-1.73)	<0.001

HBsAg, hepatitis B surface antigen; HR, hazard ratio; CI, confidence interval.

Participants with unclear or missing HBsAg terst result, or with self-reported prior cancer, liver cirrhosis, or chronic hepatitis were excluded from all analyses.

Cox models are stratified by age-at-risk, study area, and HBsAg, and adjusted for education, household income, smoking, body mass index, and physical activity.

[a]Prior chronic diseases included self-reported prior coronary heart diseases, stroke, transient ischaemic attack, tuberculosis, rheumatoid arthritis, peptic ulcer, emphysema/bronchitis, gallstone/gallbladder disease, kidney disease, and prevalent diabetes (both self-reported and screen-detected).

Table S18. Attributable fraction of liver diseases from ever-regular drinking in men in the CKB

	N	HR (95% CI)	Proportion of ever-regular drinkers	Attributable Fraction (approximate 95% CI), %
Liver cancer	1592	1.22 (1.10-1.36)	0.47	8.5 (4.3-12.7)
Liver cirrhosis	1098	1.21 (1.06-1.38)	0.46	7.9 (2.8-12.9)
Non-alcoholic fatty liver disease	440	1.26 (1.03-1.54)	0.60	12.5 (2.9-22.2)
Non-neoplastic liver diseases (all liver diseases excluding liver cancer)[a]	3529	1.21 (1.13-1.30)	0.50	8.7 (5.7-11.7)
Total liver disease	4641	1.21 (1.14-1.29)	0.49	8.7 (6.1-11.2)

HR, hazard ratio; CI, confidence interval; ICD-10, international classification of diseases, 10th revision.
Participants with unclear or missing HBsAg test result, or with self-reported prior cancer, liver cirrhosis, or chronic hepatitis were excluded.
Cox models are stratified by age-at-risk, study area, and HBsAg, and adjusted for education, household income, smoking, physical activity, and body mass index.
Attributable fraction is calculated as P(HR-1)/HR, where P is the prevalence of ever-regular alcohol consumption (i.e., current and ex-regular drinking) among those who developed the relevant liver disease during follow-up.
[a]Included all non-neoplastic liver diseases (ICD-10: B18-B19, B94.2, K70-K77, Z22.5).

Table S19. Adjusted HRs for liver disease mortality associated with drinking status, in men

	All	Abstainers		Ex-regular drinkers		Occasional drinkers		Current regular drinkers		
	N	N	HR (95% CI)	N	HR (95% CI)	N	HR (95% CI)	N	HR (95% CI)	*P* value[a]
Liver cancer	1172	272	1.00 (0.88-1.14)	152	1.28 (1.09-1.50)	358	0.87 (0.78-0.98)	390	1.07 (0.96-1.19)	0.441
Liver cirrhosis	141	32	1.00 (0.68-1.47)	21	1.03 (0.66-1.61)	36	0.65 (0.46-0.92)	52	0.84 (0.62-1.13)	0.493
Alcoholic liver disease	58	5	1.00 (0.41-2.46)	3	1.64 (0.53-5.12)	6	1.17 (0.52-2.66)	44	7.11 (5.09-9.92)	<0.001
Chronic viral hepatitis	123	39	1.00 (0.71-1.41)	9	0.82 (0.42-1.60)	42	0.82 (0.60-1.12)	33	1.00 (0.68-1.46)	0.994
Total liver disease	1525	359	1.00 (0.89-1.12)	190	1.23 (1.07-1.43)	451	0.85 (0.77-0.94)	525	1.11 (1.01-1.21)	0.182

HR, hazard ratio; CI, confidence interval; HBsAg, hepatitis B surface antigen.
Participants with unclear or missing HBsAg test result, or with self-reported prior cancer, liver cirrhosis, or chronic hepatitis were excluded.
Cox models are stratified by age-at-risk, study area, and HBsAg, and adjusted for education, household income, smoking, body mass index, and physical activity.
No deaths from non-alcoholic fatty liver disease as underlying cause were recorded.
[a]*P* for association comparing current regular drinkers vs. abstainers.

Table S20. Adjusted HRs for liver disease mortality associated with level of alcohol consumption, in male current regular drinkers

		<140 g/week		140-279 g/week		280-419 g/week		≥420 g/week		Per 280 g/week usual intake		
	All current regular N	N	HR (95% CI)	N	HR (95% CI)	N	HR (95% CI)	N	HR (95% CI)	N	HR (95% CI)	*P* trend
Liver cancer	390	105	1.00 (0.81-1.24)	92	1.18 (0.96-1.45)	89	1.62 (1.31-1.99)	104	1.90 (1.54-2.35)	390	1.54 (1.29-1.83)	<0.001
Liver cirrhosis	52	8	1.00 (0.48-2.08)	15	2.35 (1.38-3.99)	11	2.72 (1.51-4.89)	18	3.82 (2.25-6.50)	52	2.11 (1.56-2.86)	<0.001
Alcoholic liver disease	44	6	1.00 (0.42-2.36)	9	1.85 (0.95-3.61)	8	2.61 (1.31-5.17)	21	5.44 (3.33-8.87)	44	2.03 (1.56-2.62)	<0.001
Chronic viral hepatitis	33	9	1.00 (0.48-2.08)	9	1.48 (0.77-2.85)	7	1.68 (0.79-3.59)	8	1.71 (0.82-3.56)	33	1.73 (0.95-3.18)	0.075
Total liver disease	525	128	1.00 (0.83-1.21)	127	1.33 (1.12-1.59)	116	1.74 (1.45-2.09)	154	2.24 (1.88-2.66)	525	1.73 (1.52-1.96)	<0.001

HR, hazard ratio; CI, confidence interval; HBsAg, hepatitis B surface antigen.
Participants with unclear or missing HBsAg test result, or with self-reported prior cancer, liver cirrhosis, or chronic hepatitis were excluded.
Cox models are stratified by age-at-risk, study area, and HBsAg, and adjusted for education, household income, smoking, body mass index, and physical activity.
No deaths from non-alcoholic fatty liver disease as underlying cause were recorded.

References

1. Millwood IY, Li L, Smith M, Guo Y, Yang L, Bian Z, et al. Alcohol consumption in 0.5 million people from 10 diverse regions of China: prevalence, patterns and socio-demographic and health-related correlates. *Int J Epidemiol.* 2013;42(3):816-827.

2. Im PK, Millwood IY, Guo Y, Du H, Chen Y, Bian Z, et al. Patterns and trends of alcohol consumption in rural and urban areas of China: findings from the China Kadoorie Biobank. *BMC Public Health.* 2019;19(1):217.

3. Im PK, Millwood IY, Chen Y, Guo Y, Du H, Kartsonaki C, et al. Problem drinking, wellbeing and mortality risk in Chinese men: findings from the China Kadoorie Biobank. *Addiction.* 2020;115(5):850-862.

4. Im PK, Millwood IY, Kartsonaki C, Chen Y, Guo Y, Du H, et al. Alcohol drinking and risks of total and site-specific cancers in China: a 10-year prospective study of 0.5 million adults. *Int J Cancer.* 2021.

5. Cochrane J, Chen H, Conigrave KM, Hao W. Alcohol use in China. *Alcohol Alcohol.* 2003;38(6):537-542.

6. Easton DF, Peto J, Babiker AG. Floating absolute risk: an alternative to relative risk in survival and case-control analysis avoiding an arbitrary reference group. *Stat Med.* 1991;10(7):1025-1035.

7. Clarke R, Shipley M, Lewington S, Youngman L, Collins R, Marmot M, et al. Underestimation of risk associations due to regression dilution in long-term follow-up of prospective studies. *Am J Epidemiol.* 1999;150(4):341-353.

8. MacMahon S, Peto R, Cutler J, Collins R, Sorlie P, Neaton J, et al. Blood pressure, stroke, and coronary heart disease. Part 1, Prolonged differences in blood pressure: prospective observational studies corrected for the regression dilution bias. *Lancet.* 1990;335(8692):765-774.

9. Rosner B, Willett WC, Spiegelman D. Correction of logistic regression relative risk estimates and confidence intervals for systematic within-person measurement error. *Stat Med.* 1989;8(9):1051-1073.

10. Bragg F, Holmes MV, Iona A, Guo Y, Du H, Chen Y, et al. Association between diabetes and cause-specific mortality in rural and urban areas of China. *JAMA.* 2017;317(3):280-289.

11. World Health Organization. *International Guide for Monitoring Alcohol Consumption and Related Harm.* Geneva: World Health Organization; 2000.
